# Plant foods, dietary fibre and risk of ischaemic heart disease in the European Prospective Investigation into Cancer and Nutrition (EPIC) cohort

**DOI:** 10.1093/ije/dyaa155

**Published:** 2020-11-27

**Authors:** Aurora Perez-Cornago, Francesca L Crowe, Paul N Appleby, Kathryn E Bradbury, Angela M Wood, Marianne Uhre Jakobsen, Laura Johnson, Carlotta Sacerdote, Marinka Steur, Elisabete Weiderpass, Anne Mette L Würtz, Tilman Kühn, Verena Katzke, Antonia Trichopoulou, Anna Karakatsani, Carlo La Vecchia, Giovanna Masala, Rosario Tumino, Salvatore Panico, Ivonne Sluijs, Guri Skeie, Liher Imaz, Dafina Petrova, J Ramón Quirós, Sandra Milena Colorado Yohar, Paula Jakszyn, Olle Melander, Emily Sonestedt, Jonas Andersson, Maria Wennberg, Dagfinn Aune, Elio Riboli, Matthias B Schulze, Emanuele di Angelantonio, Nicholas J Wareham, John Danesh, Nita G Forouhi, Adam S Butterworth, Timothy J Key

**Affiliations:** 1 Cancer Epidemiology Unit, Nuffield Department of Population Health, University of Oxford, Oxford, UK; 2 Institute of Applied Health Research, University of Birmingham, Birmingham, UK; 3 National Institute for Health Innovation, School of Population Health, University of Auckland, Auckland, New Zealand; 4 British Heart Foundation Cardiovascular Epidemiology Unit, Department of Public Health and Primary Care, University of Cambridge, Cambridge, UK; 5 British Heart Foundation Centre of Research Excellence, University of Cambridge, Cambridge, UK; 6 National Institute for Health Research Blood and Transplant Research Unit in Donor Health and Genomics, University of Cambridge, Cambridge, UK; 7 National Institute for Health Research Cambridge Biomedical Research Centre, University of Cambridge and Cambridge University Hospitals, Cambridge, UK; 8 Medical Research Council Biostatistics Unit, Cambridge Institute of Public Health, University of Cambridge, Cambridge, UK; 9 Health Data Research UK Cambridge, Wellcome Genome Campus and University of Cambridge, Cambridge, UK; 10 The Alan Turing Institute, London, UK; 11 Department of Public Health, Aarhus University, Aarhus, Denmark; 12 Division for Diet, Disease Prevention and Toxicology, National Food Institute, Technical University of Denmark, Kgs. Lyngby, Denmark; 13 Centre for Exercise, Nutrition and Health Sciences, School for Policy Studies, University of Bristol, UK; 14 Unit of Cancer Epidemiology, Città della Salute e della Scienza University-Hospital and Center for Cancer Prevention (CPO), Turin, Italy; 15 MRC Epidemiology Unit, University of Cambridge, Cambridge, UK; 16 International Agency for Research on Cancer, World Health Organization, Lyon, France; 17 Division of Cancer Epidemiology, German Cancer Research Center (DKFZ), Heidelberg, Germany; 18 Hellenic Health Foundation, Athens, Greece; 19 2nd Pulmonary Medicine Department, School of Medicine, National and Kapodistrian University of Athens, “ATTIKON” University Hospital, Haidari, Greece; 20 Department of Clinical Sciences and Community Health Università degli Studi di Milano, Milan, Italy; 21 Cancer Risk Factors and Lifestyle Epidemiology Unit, Institute for Cancer Research, Prevention and Clinical Network (ISPRO), Florence, Italy; 22 Cancer Registry and Histopathology Department, “M.P.Arezzo” Hospital, ASP Ragusa, Italy; 23 Dipartimento Di Medicina Clinica E Chirurgia Federico Ii University, Naples, Italy; 24 Julius Center for Health Sciences and Primary Care, University Medical Center Utrecht, Utrecht University, Utrecht, the Netherlands; 25 Department of Community Medicine, UiT the Arctic University of Norway, Tromsø, Norway; 26 The Nutrition Epidemiology Group, School of Food Science and Nutrition, University of Leeds, Leeds, UK; 27 Public Health Division of Gipuzkoa, Health Department of Basque Country, Spain; 28 Andalusian School of Public Health (EASP), Granada, Spain; 29 Instituto de Investigaciœn Biosanitaria de Granada (ibs.GRANADA), Universidad de Granada, Granada, Spain; 30 CIBER de Epidemiologia y Salud Publica (CIBERESP), Madrid, Spain; 31 Public Health Directorate, Asturias, Spain; 32 Department of Epidemiology, Murcia Regional Health Council, IMIB-Arrixaca, Murcia, Spain; 33 Research Group on Demography and Health, National Faculty of Public Health, University of Antioquia, MedellÚn, Colombia; 34 Nutrition and Cancer Unit, Cancer Epidemiology Research Programme, Catalan Institute of Oncology, Barcelona, Spain; 35 Facultad de Ciencias de la salud, Universidad Ramon LLul, Barcelona, Spain; 36 Department of Clinical Sciences, Malmö, Lund University, Malmö, Sweden; 37 Department of Emergency and Internal Medicine, Skåne University Hospital, Malmö, Sweden; 38 Nutritional Epidemiology, Department of Clinical Sciences Malmö, Lund University, Malmö, Sweden; 39 Department of Public Health and Clinical Medicine, Research Unit Skellefteå, Umeå University, Umeå, Sweden; 40 Department of Public Health and Clinical Medicine, Section of Sustainable Health/Nutritional Research, Umeå University, Umeå, Sweden; 41 Department of Epidemiology and Biostatistics, School of Public Health, Imperial College London, London, UK; 42 Department of Nutrition, Bjørknes University College, Oslo, Norway; 43 Department of Endocrinology, Morbid Obesity and Preventive Medicine, Oslo University Hospital, Oslo, Norway; 44 Department of Molecular Epidemiology, German Institute of Human Nutrition Potsdam-Rehbruecke, Nuthetal, Germany; 45 Institute of Nutritional Sciences, University of Potsdam, Nuthetal, Germany; 46 Health Data Research UK Cambridge, Wellcome Genome Campus and University of Cambridge, Cambridge, UK; 47 British Heart Foundation Centre of Research Excellence, University of Cambridge, Cambridge, UK; 48 Department of Human Genetics, Wellcome Sanger Institute, Hinxton, UK

**Keywords:** Fruit, vegetables, legumes, nuts, seeds, coronary heart disease

## Abstract

**Background:**

Epidemiological evidence indicates that diets rich in plant foods are associated with a lower risk of ischaemic heart disease (IHD), but there is sparse information on fruit and vegetable subtypes and sources of dietary fibre. This study examined the associations of major plant foods, their subtypes and dietary fibre with risk of IHD in the European Prospective Investigation into Cancer and Nutrition (EPIC).

**Methods:**

We conducted a prospective analysis of 490 311 men and women without a history of myocardial infarction or stroke at recruitment (12.6 years of follow-up, *n* cases = 8504), in 10 European countries. Dietary intake was assessed using validated questionnaires, calibrated with 24-h recalls. Multivariable Cox regressions were used to estimate hazard ratios (HR) of IHD.

**Results:**

There was a lower risk of IHD with a higher intake of fruit and vegetables combined [HR per 200 g/day higher intake 0.94, 95% confidence interval (CI): 0.90–0.99, *P*-trend* *=* *0.009], and with total fruits (per 100 g/day 0.97, 0.95–1.00, *P-*trend* *=* *0.021). There was no evidence for a reduced risk for fruit subtypes, except for bananas. Risk was lower with higher intakes of nuts and seeds (per 10 g/day 0.90, 0.82–0.98, *P-*trend* *=* *0.020), total fibre (per 10 g/day 0.91, 0.85–0.98, *P-*trend* *=* *0.015), fruit and vegetable fibre (per 4 g/day 0.95, 0.91–0.99, *P-*trend* *=* *0.022) and fruit fibre (per 2 g/day 0.97, 0.95–1.00, *P-*trend* *=* *0.045). No associations were observed between vegetables, vegetables subtypes, legumes, cereals and IHD risk.

**Conclusions:**

In this large prospective study, we found some small inverse associations between plant foods and IHD risk, with fruit and vegetables combined being the most strongly inversely associated with risk. Whether these small associations are causal remains unclear.

**Key Messages**

- Epidemiological evidence indicates that diets rich in plant foods are associated with a moderately lower risk of ischaemic heart disease (IHD), but there is sparse information on fruit and vegetable subtypes and sources of dietary fibre.
- Our study found that higher intakes of fruit and vegetables combined, total fruit, bananas, nuts and seeds, total fibre, fruit and vegetable combined fibre and fruit fibre are associated with a lower risk of IHD, of small magnitude.
- To the best of our knowledge, this is the largest prospective study looking at major plant foods, their subtype, and dietary fibre in relation to IHD risk including incident IHD cases and death from IHD.
- As with other observational studies, the associations reported may be subject to residual confounding, and whether these small associations are causal remains unclear.

## Introduction

Ischaemic heart disease (IHD) is the leading cause of death worldwide,^1^ and its prevention is an urgent public health priority. Plant-based diets rich in fruit, vegetables, legumes and nuts and seeds have been associated with a lower risk of IHD, possibly because of their effect on blood pressure, blood lipids or other cardiovascular risk factors, although it is also possible that these associations are due to residual confounding by other risk factors.^2–5^ There are a number of nutrients in fruits, vegetable and legumes that could be responsible for providing protection against IHD, including potassium, flavonoids and dietary fibre.^6–10^ Nuts and whole seeds are good sources of fibre, and they also contain unsaturated fatty acids, magnesium and other potentially bioactive compounds that may perhaps provide a protective effect against IHD.^11^ However, questions remain about the associations of subtypes of fruit, vegetables and different sources of fibre with IHD risk.^2^^,^^7^

A recent meta-analysis of prospective studies found that several individual types of fruits and vegetables were inversely associated with risk of IHD (apples/pears, citrus fruits, cruciferous vegetables, green leafy vegetables, tomatoes and beta-carotene- rich and vitamin C-rich fruit and vegetables).^2^ However, due to the low number of studies reporting results on fruit and vegetable subtypes, the potential for selective reporting, and publication of subtypes that are significantly associated with risk, more prospective studies are needed.^2^

The aim of this study was to examine the associations of major plant foods (i.e. fruit, vegetables, legumes, nuts and seeds, cereals), their subtypes, and different sources of dietary fibre with the risk of new-onset IHD [including 8504 myocardial infarction (MI) cases or deaths from IHD] using data from the European Prospective Investigation into Cancer and Nutrition (EPIC) study.

## Methods

### Study population

EPIC is a prospective study examining lifestyle factors and health in approximately 520 000 men and women recruited through 23 centres in 10 European countries (Denmark, France, Germany, Greece, Italy, The Netherlands, Norway, Spain, Sweden and the UK), mostly between 1992 and 2000.^12^^,^^13^ Participants, mostly aged 35–70 years, completed dietary and lifestyle questionnaires and the majority also provided blood samples and had their blood pressure measured. All participants gave written informed consent and the study protocol was approved by ethical review boards of all institutions where participants were recruited.

In the current analyses we included data from the full EPIC cohort and from the EPIC-cardiovascular disease (EPIC-CVD) case-cohort study, which is nested within EPIC and has biomarker data.^13^^,^^14^ For the full EPIC cohort, data were available for 490 311 participants after applying the exclusion criteria (see Supplementary Methods, available as Supplementary data at *IJE* online). Data from 16 425 participants from the EPIC-CVD sub-cohort were used to assess the associations between the exposures and selected biomarkers. The sub-cohort was randomly selected from among participants with a stored blood sample, with selection stratified by the 23 EPIC recruitment centres.

### Dietary and lifestyle assessment

Dietary intake during the year before enrolment was measured using centre- or country-specific validated food frequency questionnaires (FFQs) or diet histories, as previously described.^12^ In order to improve the comparability of dietary data across the participating centres, an 8% stratified random sample across all centres also completed a standardized, computerized 24-h recall that was administered face-to-face with participants soon after enrolment into the cohort.^15^ Details of the categorization of foods are available in the Supplementary Methods and have also been described elsewhere.^16^^,^^17^ Assessments of the non-dietary variables (e.g. lifestyle, health status, sociodemographic characteristics, anthropometry and medical history) were generally based on answers in the baseline questionnaire.

### Blood samples and blood pressure

Total cholesterol and high-density lipoprotein (HDL) cholesterol were measured in stored serum and HbA_1c_ in erythrocytes as part of the EPIC-CVD case-cohort study, which is nested within EPIC.^13^ Trained health professionals measured blood pressure using a variety of devices and methods, as described in detail elsewhere.^13^^,^^18^ Details are in the Supplementary Methods.

### Ascertainment and verification of cases of ischaemic heart disease

The outcome examined in this study was IHD, defined as the first non-fatal MI (ICD-10 I21) or death from IHD (ICD-10 I20-25). Incident non-fatal MI was ascertained in each EPIC centre using a combination of self-report and record linkage with morbidity/hospital registries.^13^ Information on vital status was collected from mortality registries at the regional or national level, except in Greece where vital status was ascertained by active follow-up of study participants and next of kin. The last year of follow-up ranged between 2003 and 2010.

### Statistical analyses

Baseline characteristics of the study population were calculated separately for all participants and cases and also for men and women, and presented as means with standard deviations (SDs) for continuous variables or percentages for categorical variables.

Analyses of the association of dietary intakes of vegetables (total, fruiting, leafy, cruciferous and root vegetables), fruit (total, citrus, hard and bananas), legumes, nuts and seeds, cereals and dietary fibre (total, fruit, vegetable and cereal fibre) with first non-fatal MI or fatal IHD risk were conducted using Cox proportional hazards regression, and hazard ratios (HRs) and 95% confidence intervals (CIs) were calculated. Follow-up was analysed from recruitment until the date of first non-fatal MI or fatal IHD event, or censoring at the date of death from other causes, non-fatal non-MI IHD, the date at which follow-up for IHD events was considered complete, or emigration or other loss to follow-up (1.3%). Participants’ intakes were categorized as follows: (i) into fifths of observed intake of each food based on their responses to the recruitment FFQ; (ii) for continuous analyses, as the approximate difference in mean intake between participants in the lowest and highest fifths of observed intake and based on previous EPIC studies^16^; (iii) fruit and vegetable intakes were further analysed as the following categorical variables <5 vs ≥5 or <3, 3–4, 5–7, ≥8 servings per day, and tests for linear trend were performed using a pseudo-continuous variable using the median values in each ﬁfth of intake.

All analyses were stratified by sex and EPIC centre and adjusted for age at recruitment, smoking status, history of diabetes, previous hypertension, previous hyperlipidaemia, physical activity, employment status, level of education completed, alcohol consumption, body mass index (BMI) and observed intakes of total energy, red and processed meat and cheese—foods that have been previously related to IHD risk in this cohort (red and processed meat, and cheese).^19^

In further analyses conducted to improve the comparability of dietary data across participating centres and to correct for possible measurement error, the dietary data from the 24-h recalls were used to provide statistically calibrated estimates of dietary intakes for all individuals in the full cohort, and HRs were calculated for increments (as previously explained) in observed and calibrated intakes of each food.

The adjusted models were further mutually adjusted in sensitivity analyses for the other food groups analysed, to determine to what extent the associations between the plant foods and IHD risk were independent of the other relevant food groups. We also adjusted the main fully adjusted model for hormone replacement therapy in women. Sensitivity analyses were performed by repeating the analyses after excluding the first 4 years of follow-up, and we also conducted separate analyses for subsets of sex, smoking status, age at recruitment, BMI, European region and previous disease.

To explore whether the intakes of these exposures are associated with major established physiological IHD risk factors at baseline, we examined the associations of food intakes with BMI, systolic and diastolic blood pressure, total cholesterol, high-density lipoprotein (HDL) cholesterol, non-HDL cholesterol and glycated haemoglobin (HbA_1c_) (restricted to participants with these measurements in the sub-cohort), calculating mean levels of these biomarkers in each fifth of dietary intake of the exposure of interest, with adjustment for age, sex and EPIC centre.

All analyses were performed using Stata version 14.0 (Stata Corporation, College Station, TX, USA), all tests of significance were two-sided, and a *P*-value less than 0.05 was considered statistically significant. Details are in the Supplementary Methods.

## Results

After an average of 12.6 years of follow-up, 8504 participants were diagnosed with non-fatal MI (*n* = 6485) or fatal IHD (*n* = 2019) among the 490 311 participants included in this study. Mean age at recruitment was 51.2 years (SD 9.9) and the mean BMI was 25.5 kg/m^2^ (SD 4.3). Cases were on average 6 years and 10 years older in men and women, respectively, compared with the full cohort. Cases were also less likely to be highly educated and be physically active and more likely to smoke or be unemployed, diabetic or hypertensive. Cases had lower consumption of fruit, vegetables, legumes, nuts and seeds, cereals and dietary fibre. Among all participants, women consumed more fruit, vegetables, legumes and nuts and seeds than men, but they consumed less cereals and total dietary fibre (Table 1). Intakes by country can be found in Supplementary Table S1, available as Supplementary data at *IJE* online.

**Table 1:** Participant characteristics at recruitment by sex and case status for any first fatal IHD or non-fatal MI (Whole EPIC cohort)

Characteristic	Men		Women	
	All men	Men cases	All women	Women cases
Number of participants (%)	140 062 (28.6)	5587 (1.1)	350 249 (71.4)	2917 (0.6)
Age at recruitment, years	52.0 (10.1)	58.0 (8.2)	50.9 (9.8)	60.4 (8.5)
BMI, kg/m^2^	26.6 (3.7)	27.3 (3.7)	25.1 (4.4)	27.0 (4.7)
Alcohol in current drinkers, g/day	21.3 (23.1)	19.2 (22.0)	8.9 (11.9)	7.5 (11.1)
Not current alcohol drinker, *n* (%)	6282 (4.5)	326 (5.8)	39 929 (11.4)	413 (14.2)
**Smoking status and cigarettes/day,^a^*n* (%)**				
Never smoker	46 872 (33.9)	1246 (22.5)	194 390 (56.8)	1211 (41.8)
Former smoker	50 140 (36.3)	1947 (35.2)	79 405 (23.2)	612 (21.1)
Current smoker, <10 or number cigarettes unknown	14 179 (10.3)	702 (12.7)	19 525 (5.7)	224 (7.7)
Current smoker, 10-19	10 430 (7.6)	622 (11.2)	28 394 (8.3)	501 (17.3)
Current smoker, ≥20	16 471 (11.9)	1013 (18.3)	20 306 (5.9)	347 (12.0)
**Highest level of education completed,^a^*n* (%)**				
None or primary	46 534 (34.1)	2508 (46.3)	98 232 (29.2)	1418 (52.1)
Secondary	18 464 (13.5)	523 (9.7)	82 970 (24.7)	252 (9.3)
Vocational or university	71 292 (52.3)	2386 (44.0)	155 097 (46.1)	1053 (38.7)
**Cambridge physical activity index,^a^*n* (%)**				
Inactive	25 881 (18.9)	1415 (25.8)	76 462 (22.2)	984 (34.3)
Moderately inactive	42 888 (31.3)	1701 (31.1)	119 269 (34.7)	966 (33.7)
Moderately active	34 208 (25.0)	1213 (22.2)	95 575 (27.8)	523 (18.2)
Active	34 022 (24.8)	1146 (20.9)	52 887 (15.4)	394 (13.7)
**Employed or student,^a^*n* (%)**				
Yes	94 149 (75.9)	3001 (60.1)	208 655 (65.5)	1046 (38.6)
No	29 813 (24.1)	1990 (39.9)	109 816 (34.5)	1665 (61.4)
**History of diabetes,^a^*n* (%)**				
No	132 471 (96.6)	4982 (92.6)	328 568 (97.8)	2533 (91.6)
Yes	4640 (3.4)	399 (7.4)	7260 (2.2)	232 (8.4)
**Previous hypertension,^a^*n* (%)**				
No	107 220 (79.9)	3771 (71.0)	273 692 (82.1)	1757 (62.8)
Yes	26 926 (20.1)	1538 (29.0)	59 856 (17.9)	1040 (37.2)
**Region,^b^*n* (%)**				
Northern Europe	46 911 (33.5)	2992 (53.6)	93 893 (26.8)	1395 (47.8)
Central Europe	53 624 (38.3)	1556 (27.9)	164 338 (46.9)	1079 (37.0)
Southern Europe	39 527 (28.2)	1039 (18.6)	92 018 (26.3)	443 (15.2)
**Energy intake, kcal/day**	2416 (662)	2380 (654)	1932 (540)	1856 (515)
**Foods, g/day, medians (quartiles)**				
Fruit and vegetables	328 (203, 521)	296 (186, 462)	415 (274, 593)	369 (247, 525)
Total vegetables	151 (94, 248)	139 (84, 221)	185 (118, 286)	159 (102, 244)
Fruiting vegetables^c^	46 (25, 83)	37 (20, 69)	57 (31, 96)	46 (25, 79)
Leafy vegetables^d^	10 (2, 29)	5 (1, 20)	21 (7, 53)	10 (2, 27)
Cruciferous vegetables^e^	13 (4, 30)	12 (3, 29)	18 (6, 39)	15 (4, 36)
Root vegetables	12 (5, 29)	12 (4, 30)	21 (8, 42)	19 (7, 41)
Fruit	156 (82, 280)	140 (71, 249)	209 (120, 323)	190 (114, 297)
Citrus fruit	21 (8, 62)	17 (6, 47)	36 (9, 72)	32 (9, 69)
Apples and pears	44 (13, 102)	43 (11, 105)	46 (15, 97)	57 (19, 110)
Bananas	11 (2, 38)	10 (2, 38)	11 (1, 40)	15 (4, 43)
Legumes^f^	8.0 (1.5, 23.1)	6.2 (0.0, 20.7)	9.8 (1.5, 23.1)	5.0 (0.0, 14.3)
Nuts and seeds^e^	0.8 (0.1, 4.3)	0.7 (0.0, 2.5)	1.1 (0.0, 4.3)	0.5 (0.0, 1.9)
Cereals and cereal products	236 (171, 316)	218 (154, 295)	187 (135, 251)	160 (114, 219)
**Other foods, g/day, medians (quartiles)**				
Red and processed meat	90 (54, 131)	96 (61, 139)	60 (35, 90)	65 (41, 93)
Cheese	28 (15, 52)	24 (13, 49)	30 (16, 54)	23 (13, 41)
**Dietary fibre, g/day, medians (quartiles)**				
Total dietary fibre	23.3 (18.4, 29.1)	22.4 (17.3, 28.0)	21.3 (17.0, 26.1)	19.9 (15.7, 25.0)
Fruit and vegetable fibre	6.8 (4.4, 10.2)	6.3 (3.9, 9.4)	8.5 (5.8, 11.8)	7.7 (5.2, 10.6)
Vegetable fibre	3.5 (2.2, 5.5)	3.2 (1.9, 5.0)	4.3 (2.8, 6.3)	3.7 (2.3, 5.4)
Fruit fibre	2.8 (1.5, 4.8)	2.6 (1.3, 4.5)	3.8 (2.2, 5.9)	3.5 (2.1, 5.6)
Cereal fibre	9.5 (6.4, 13.7)	9.4 (6.2, 13.8)	7.1 (4.8, 10.3)	7.2 (4.9, 10.1)

Values are means (SD) except where otherwise indicated. Percentages do not match due to missing data.

a Value or category unknown for some participants.

b Northern Europe: Denmark, Norway, Sweden; Central Europe: France excepting Provence and SW France, Germany, The Netherlands, UK; Southern Europe: Greece, Italy, Spain, Provence, SW France.

c Such as tomatoes, cucumbers and sweet peppers; unavailable for Norway.

d Unavailable for Norway and Umea.

e Unavailable for Umea.

f Unavailable for Denmark and Norway.

The risk of IHD by the number of standard portions of fruits and vegetables consumed per day is shown in Table 2. Participants consuming at least five portions of fruits and vegetables a day had a lower risk of IHD (HR for ≥8 portions/day 0.90, 95% CI: 0.82–0.98; HR for ≥5 to <8 portions/day 0.92, 0.86–0.98) compared with those consuming fewer than three portions a day. Vegetable intake was not significantly related to IHD risk, whereas fruit intake was related to a reduced risk (HR for ≥2.5-<4 portions/day 0.92, 0.86-0.98; HR for ≥4 portions/day 0.92, 0.85–0.98 compared with those consuming <1.5 portions/day).

**Table 2:** Hazard ratios (95% confidence intervals) for any first fatal IHD or non-fatal MI per portions per day of fruit and vegetables (whole EPIC cohort)

Food group	Portions/day			
Fruit and vegetables	<5	≥5		
Adjusted HR (95% CI)	1 ref	0.93 (0.88–0.98)		
Fruit and vegetables	<3	≥3–<5	≥5–<8	≥8
Number of IHD cases	2811	2624	2000	1069
Adjusted HR (95% CI)	1 ref	0.97 (0.92–1.03)	0.92 (0.86–0.98)	0.90 (0.82–0.98)
Vegetables	<1.5	≥1.5–<2.5	≥2.5–<4	≥4
Number of IHD cases	3315	2509	1656	1024
Adjusted HR (95% CI)	1 ref	1.01 (0.95–1.06)	0.93 (0.87–1.00)	0.96 (0.88–1.05)
Fruit	<1.5	≥1.5–<2.5	≥2.5–<4	≥4
Number of IHD cases	3204	2034	1765	1501
Adjusted HR (95% CI)	1 ref	0.98 (0.92–1.03)	0.92 (0.86–0.98)	0.92 (0.85–0.98)

Hazard ratios are adjusted for age (continuous), smoking status and number of cigarettes per day (never smoker, former smoker, current smoker <10 cigs/day, current smoker 10–19 cigarettes/day, current smoker 20+ cigarettes/day, unknown), histories of diabetes, hypertension and hyperlipidaemia (each yes, no, unknown), Cambridge physical activity index (inactive, moderately inactive, moderately active, active, unknown), employment status (employed or student, not employed or student, unknown), level of education completed (none or primary, secondary, vocational or university, unknown), current alcohol consumption (non–drinkers and sex–specific fifths of intake among drinkers), BMI (<22.5, 22.5–24.9, 25.0–27.4, 27.5–29.9, ≥30.0 kg/m^2^, unknown), and observed intakes of total energy, red and processed meat, and cheese (each continuous), and stratified by sex and EPIC centre.

**Figure 1:**
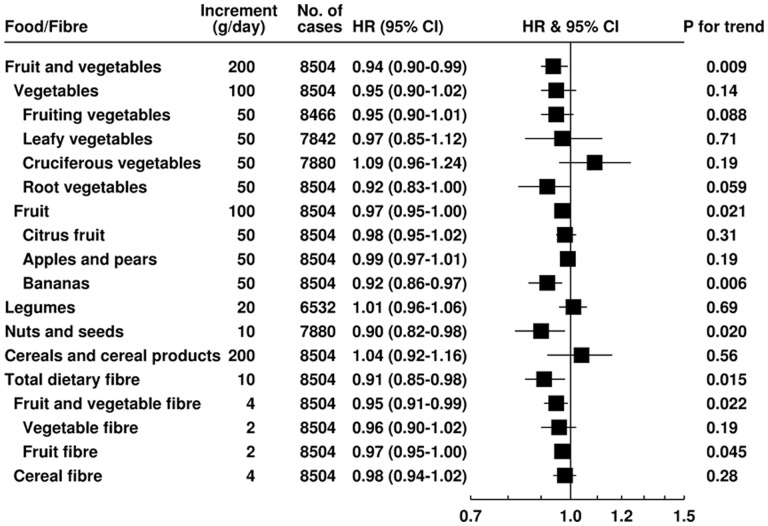
Hazard ratios (95% CI) for first non-fatal MI and fatal IHD per increment in statistically calibrated intake of plant foods and dietary fibre (Whole EPIC cohort). Hazard ratios are adjusted for age (continuous), smoking status and number of cigarettes per day (never smoker, former smoker, current smoker (10 cigs/d, current smoker 10-19 cigs/d, current smoker 20+ cigs/d, unknown), histories of diabetes, hypertension and hyperlipidaemia (each yes, no, unknown), Cambridge physical activity index (inactive, moderately inactive, moderately active, active, unknown), employment status (employed or student, not employed or student, unknown), level of education completed (none or primary, secondary, vocational or university, unknown), current alcohol consumption (non-drinkers and sex-specific fifths of intake among drinkers), BMI (h22.5, 22.5-24.9, 25.0-27.4, 27.5-29.9, 30.0 kg/m2, unknown), and calibrated intakes of total energy, red and processed meat, and cheese (each continuous), and stratified by sex and EPIC centre. Tests of trend were performed using the calibrated intake. Fruiting vegetables, such as tomatoes, cucumbers and sweet peppers; unavailable for Norway. Leafy vegetables were unavailable for Norway and Umea. Cruciferous vegetables and nuts and seeds intakes were not available for Umea. Legumes were unavailable for Denmark and Norway. Abbreviations: Body mass index (BMI), ischaemic heart disease (IHD), myocardial infarction (MI).

The associations of IHD with intakes of fruit, vegetables, legumes, nuts and seeds, cereals and fibre, per increment approximately equivalent to the difference in mean intake per day between participants in the lowest and highest fifths of intake, are shown in Figure 1 (for calibrated intake) and Supplementary Table S2, available as Supplementary data at *IJE* online (for observed intake, including associations by fifths of dietary intakes). Each 200 g/day higher intake of fruit and vegetables combined was associated with a 6% lower risk of IHD (HR 0.94, 95% CI: 0.90–0.99, *P-*trend =* *0.009). Total vegetables, as well as vegetable subtypes, were not significantly related to IHD risk. Each 100-g/day higher intake of total fruit intake was associated with a 3% lower risk of IHD (0.97, 0.95–1.00, *P-*trend* *=* *0.021); however, among individual fruit subgroups only higher intake of bananas was associated with lower risk (HR for each 50 g/da 0.92, 0.86–0.97, *P-*trend = 0.006). Legume and cereal consumption were not related to IHD risk, whereas a 10-g/day higher intake of nuts and seeds was associated with a 10% lower risk of IHD (0.90, 0.82–0.98, *P-*trend =* *0.020). Associations between nuts and seeds and IHD risk were similar when a 5-g/day increment was used (0.95, 0.01–0.99, *P-*trend* *=* *0.020); 10-g/day higher total fibre intake was associated with a 9% lower risk of IHD (0.91, 0.85–0.98, *P-*trend* *=* *0.015). Considering different fibre sources, each 4-g/day higher intake of fruit and vegetable fibre was associated with a 5% lower risk of IHD (0.95, 0.91–0.99, *P-*trend* *=* *0.022), and each 2-g/day higher intake of fibre from fruit was related to a 3% lower IHD risk (0.97, 0.95–1.00, *P-*trend =* *0.045). Higher intakes of fibre from other sources showed no clear associations with risk of IHD.

There were no significant differences when the multivariable adjusted model was mutually adjusted for other plant foods, or when it was adjusted for hormone replacement therapy in women (Supplementary Table S3, available as Supplementary data at *IJE* online). After excluding the first 4 years of follow-up, the 95% confidence intervals were wider and only bananas and nuts and seeds remained significantly associated with IHD risk (Supplementary Table S4, available as Supplementary data at *IJE* online). There was no evidence of heterogeneity by smoking status or age at recruitment (Supplementary Tables S5 and S6, available as Supplementary data at *IJE* online). There was some evidence of heterogeneity by sex for leafy vegetables (*P*-heterogeneity* *=* *0.018), although there were no significant associations with IHD for either men or women (Supplementary Table S7, available as Supplementary data at *IJE* online). There was no evidence of heterogeneity by BMI, European region or previous disease (Supplementary Tables S8–S10, available as Supplementary data at *IJE* online).

Compared with participants in the lowest fifths of fruit and vegetables combined and dietary fibre intakes, those in the highest fifths of intake had lower systolic (by 1.0 and 1.2 mmHg, respectively) and diastolic blood pressure (by 0.4 and 1.0 mmHg, respectively) and non-HDL cholesterol concentrations (0.16 and 0.14 mmol/L, respectively) (Supplementary Table S11, available as Supplementary data at *IJE* online). Participants in the highest fifth of fruit and vegetables intake had higher BMI (0.33 kg/m^2^), whereas those in the highest fifth of dietary fibre had lower BMI (−0.20 kg/m^2^). Similar associations were observed when fruit and vegetables were assessed separately. Participants in the highest fifth of nuts and seeds intake had a lower BMI and blood pressure, and they also had higher HDL cholesterol and lower non-HDL cholesterol (Supplementary Table S11, available as Supplementary data at *IJE* online).

## Discussion

In this large European cohort, we found some small inverse associations between plant foods and IHD risk, with fruit and vegetables combined being the most strongly inversely associated with risk. As far as we are aware this is the largest prospective study looking at major plant foods, their subtypes and dietary fibre in relation to IHD risk including incident IHD cases and death from IHD.

The most recent meta-analysis of prospective studies on fruit and vegetables intake and IHD risk, which included up to 25 prospective studies, reported that the relative risk (RR) of IHD for each 200-g/day intake of fruit and vegetables combined was 8% lower,^2^ which is similar to the results from our study; a subsequent large prospective study including data from seven geographical world regions also showed a lower risk of IHD with a higher consumption of fruit and vegetables.^20^ Whereas results from the meta-analysis also showed that intakes of both fruit and vegetables separately were inversely related to a lower IHD risk,^2^ our study showed that only the intake of fruit was significantly associated with a lower risk. However, the association of fruit intake with IHD risk was weaker in our study (3% lower risk, per 100-g/day higher intake) than in this meta-analysis (10% lower risk, per 200 g/day).^2^ Regarding fruit subtypes, only bananas were associated with a lower IHD risk in our study; however, results from a meta-analysis including only three prospective studies showed no association of bananas with IHD risk.^2^

The small inverse association between fruit and vegetables and risk of IHD, if causal, might be at least partly mediated through blood pressure and non-HDL cholesterol concentrations, which are well-established IHD risk factors; in our EPIC-CVD sub-cohort, participants in the highest fifth of fruit intake had modestly lower systolic blood pressure and non-HDL cholesterol concentrations than those in the lowest fifth. However, there is no evidence from large randomized controlled trials on the effect of fruit consumption alone on blood pressure although some,^21–23^ but not all,^24^ randomized controlled trials where participants have increased their consumption of fruits and vegetables combined have shown a reduction in blood pressure. It is also possible that serum non-HDL cholesterol might mediate some of the effect of fruit intake on IHD risk, although evidence from randomized controlled trials does not support this hypothesis.^24^^,^^25^ There might be other mechanisms through which fruit could exert an effect on IHD risk, such as lowering oxidative stress or reducing low-grade inflammation.^26^

Legume consumption was not related to IHD risk in our study, whereas a meta-analysis of prospective studies has shown a modest and borderline significant inverse association between legume intake and IHD risk based on 10 prospective studies (RR for comparing the highest with the lowest categories of legume intake: 0.91, 95% CI 0.84-0.99).^4^ Randomized controlled trials have shown that increased legume consumption can decrease total cholesterol and non-HDL cholesterol, and suggested that several compounds in legumes, such as fibre and some bioactive compounds, may underlie this association.^27^^,^^28^

In the current study, nuts and seeds consumption was weakly inversely related to IHD risk; this association was significant in analyses assessing per increment difference in intakes, but not when we compared the highest with the lowest fifth. Results from a meta-analysis of prospective studies^29^ and from a recent large prospective study^3^ have reported that nut consumption is associated with lower IHD risk. Nuts and seeds are high in dietary fibre, unsaturated fatty acids, vitamins, minerals and several bioactive compounds that may provide a protective effect against IHD.^11^ Higher levels of consumption of nuts and seeds were related to lower BMI and blood pressure, and also with slightly higher HDL-cholesterol and lower non-HDL-cholesterol, in our EPIC-CVD sub-cohort, and meta-analyses of randomized trials have shown that nut consumption reduces total cholesterol, LDL cholesterol and apolipoprotein B.^30^ In our study, mean nut consumption was less than 1 g/day, and the inverse association we observed was for an estimated increment of only 5 or 10 g/day, which is a small quantity, and may perhaps suggest that the inverse association with risk might be due to an overall dietary pattern, or non-dietary confounders, rather than the nuts themselves.

Cereals and cereal fibre were not related to IHD risk in our study. A meta-analysis of prospective studies found that higher wholegrain intake was related to lower IHD risk (RR = 0.81, 95% CI 0.75–0.87)^31^; however, we could not isolate wholegrains in our study because some of the FFQs used in this cohort did not differentiate between types of cereals.

Previous studies have suggested that the source or type of dietary fibre may be relevant for IHD.^32^^,^^33^ In this study, we found inverse associations between the intakes of total fibre, fruit and vegetable fibre, and fruit fibre, and risk of IHD. Evidence from a recent meta-analysis of 6449 IHD deaths from nine prospective studies showed a 19% lower risk of IHD (0.81, 0.73-0.90) per 8-g/day higher intake of total dietary fibre.^34^ Our results, which include a greater number of cases, showed a weaker association between dietary fibre and incident IHD. The potential mechanisms through which fibre may protect against IHD include binding bile acids and/or dietary fats (including saturated fat) in the intestinal lumen, which may reduce circulating cholesterol levels.^32^^,^^35^ Indeed, we observed that participants in the EPIC-CVD sub-cohort who consume more fibre have slightly lower total and non-HDL cholesterol.

Strengths of this study include its prospective design, the large number of verified IHD cases and the dietary diversity of the study population across 10 countries in Europe. Moreover, the dietary questionnaires were validated in all EPIC centres and calibrated using values of intake of plant foods taken from a standardized measure of diet (24-h recall) to correct for over- and under-estimation of dietary intake.^36^

Our study also had some limitations. Although we adjusted for multiple confounders, potential unmeasured and residual confounding could still affect the observed associations. Dietary information was collected up to 12 years before IHD diagnosis or death, and consumption may have changed during follow-up. In addition, some of the associations observed might be due to chance because of the number of tests performed. The importance of the results should also be interpreted in relation to the strength of the associations.^37^ In this sense, our HR of 0.94 for fruit and vegetables combined is small compared with other risk factors, and therefore confounding by other factors cannot be excluded. Moreover, the EPIC study does not have information on whether vegetables were consumed raw or cooked, which may affect the nutrient content and bioavailablity. The average consumption of fruit and vegetables in this cohort was equivalent to about five 80-g portions a day, which is slightly higher than that of the general population in European countries such as the UK,^38^ The Netherlands^39^ and Germany.^40^

In conclusion, we found some small inverse associations between plant foods and IHD risk, with fruit and vegetables combined being the most strongly inversely associated with risk. Although the associations found in this study were not strong and could be affected by residual confounding, our results are in line with overall recommendations to increase intake of plant-based foods.

## Supplementary data

Supplementary data are available at *IJE* online.

## Funding

Analyses were supported by the gUK Medical Research Council (MR/M012190/1), Cancer Research UK (C8221/A19170 and 570/A16491) and the Wellcome Trust (Our Planet Our Health, Livestock Environment and People 205212/Z/16/Z). A.P.C. is supported by a Cancer Research UK Population Research Fellowship (C60192/A28516) and by the World Cancer Research Fund (WCRF UK), as part of the Word Cancer Research Fund International grant programme (2019/1953). EPIC-CVD has been supported by the European Union Framework 7 (HEALTH-F2-2012–279233), the European Research Council (268834), the UK Medical Research Council (G0800270 and MR/L003120/1), the British Heart Foundation (SP/09/002 and RG/08/014 and RG13/13/30194) and the UK National Institute of Health Research. The InterAct project was funded by the EU FP6 programme (grant number LSHM_CT_2006_037197) and provided the biomarker data in the sub-cohort that was used in the current study. The coordination of EPIC is financially supported by the European Commission (DG-SANCO) and the International Agency for Research on Cancer. The national cohorts are supported by: Danish Cancer Society (Denmark); Ligue Contre le Cancer, Institut Gustave Roussy, Mutuelle Générale de l’Education Nationale, Institut National de la Santé et de la Recherche Médicale (INSERM) (France); German Cancer Aid, German Cancer Research Center (DKFZ), Federal Ministry of Education and Research (BMBF) (Germany); the Hellenic Health Foundation (Greece); Associazione Italiana per la Ricerca sul Cancro-AIRC-Italy and National Research Council (Italy); Dutch Ministry of Public Health, Welfare and Sports (VWS), Netherlands Cancer Registry (NKR), LK Research Funds, Dutch Prevention Funds, Dutch ZON (Zorg Onderzoek Nederland), World Cancer Research Fund (WCRF); Health Research Fund (FIS), PI13/00061 to Granada, PI13/01162 to EPIC-Murcia, Regional Governments of Andalucía, Asturias, Basque Country, Murcia (no. 6236) and Navarra, ISCIII RETIC (RD06/0020) (Spain); Swedish Cancer Society, Swedish Research Council and County Councils of Skåne and Västerbotten (Sweden); Cancer Research UK (14136 to EPIC-Norfolk; C570/A16491 and C8221/A19170 to EPIC-Oxford), UK Medical Research Council [1000143 to EPIC-Norfolk, MR/M012190/1 to EPIC-Oxford, MC_UU_12015/1 (N.J.W.), MC_UU_12015/5 (NGF), and MC_UU_12015/520], and NIHR Biomedical Research Centre Cambridge: Nutrition, Diet, and Lifestyle Research Theme (IS-BRC-1215–20014) to the MRC Epidemiology Unit Cambridge (N.J.W., N.G.F.). J.D. holds a British Heart Foundation Chair and an NIHR Senior Investigator Award. K.E.B. holds a Girdlers’ New Zealand Health Research Council Fellowship. J.D. is funded by the National Institute for Health Research [Senior Investigator Award] [*]. A.W is supported by a BHF-Turing Cardiovascular Data Science Award and by the EC-Innovative Medicines Initiative (BigData@Heart). M.S. reports funding from the Alpro Foundation whilst at the Cardiovascular Epidemiology Unit (up to 15-08-2016), and Core MRC Unit support through the Nutritional Epidemiology Programme (MC_UU_12015/5) whilst at the MRC Epidemiology Unit (15-08-2016 until now), during the conduct of the study.

## Author contributions

Study conception and design: A.P-C, F.C., T.K. Manuscript writing: A.P-C, F.C. Data analysis: A.P-C, P.A. Interpretation of the data and critical revision and editing of the manuscript: all authors. All authors read and approved the final manuscript.

## Supplementary Material

dyaa155_Supplementary_Data
