# Supplementary material for: Plant foods, dietary fibre and risk of ischaemic heart disease in the European Prospective Investigation into Cancer and Nutrition (EPIC) cohort
Source: Int J Epidemiol. 2020 Nov 27;50(1):212–22. doi: 10.1093/ije/dyaa155 (PMC7938513; doi:10.1093/ije/dyaa155)
Supplement: dyaa155_Supplementary_Data [file dyaa155_supplementary_data.docx]

**Supplementary Material**

[**Supplementary Methods** 2](#_Toc46403924)

[**Supplementary Tables** 12](#_Toc46403925)

[**Supplementary Table S1.** Observed intakes of foods and fibre in each EPIC country. 12](#_Toc46403926)

[**Supplementary Table S2.** Hazard ratios^1^ (95% confidence intervals) for any first fatal IHD or non-fatal MI by overall fifths and increments of observed intake in plant foods and dietary fibre (Whole EPIC cohort). 13](#_Toc46403927)

[**Supplementary Table S3**. Hazard ratios^1^ (95% confidence intervals) for any first fatal IHD or non-fatal MI per increment in calibrated intake of fruit, vegetables and fibre, after further mutual adjustment of significant foods or hormone replacement therapy (Whole EPIC cohort). 16](#_Toc46403928)

[**Supplementary Table S4**. Hazard ratios^1^ (95% confidence intervals) for any first fatal IHD or non-fatal MI per increment in calibrated intake of fruit, vegetables and fibre excluding the first 4 years of follow-up (Whole EPIC cohort). 17](#_Toc46403929)

[**Supplementary Table S5**. Hazard ratios^1^ (95% confidence intervals) for first non-fatal MI or fatal IHD per increment in calibrated intake of plant foods and fibre subdivided by smoking status (Whole EPIC cohort). 18](#_Toc46403930)

[**Supplementary Table S6**. Hazard ratios^1^ (95% confidence intervals) for first non-fatal MI or fatal IHD per increment in calibrated intake of plant foods and fibre subdivided by age at recruitment (Whole EPIC cohort). 19](#_Toc46403931)

[**Supplementary Table S7.** Hazard ratios^1^ (95% confidence intervals) for first non-fatal MI or fatal IHD per increment in calibrated intake of plant foods and fibre subdivided by sex (Whole EPIC cohort). 20](#_Toc46403932)

[**Supplementary Table S8**. Hazard ratios^1^ (95% confidence intervals) for first non-fatal MI or fatal IHD per increment in calibrated intake of plant foods and fibre subdivided by BMI category (Whole EPIC cohort). 21](#_Toc46403933)

[**Supplementary Table S9.** Hazard ratios^1^ (95% confidence intervals) for first non-fatal MI or fatal IHD per increment in calibrated intake of plant foods and fibre subdivided by European region (Whole EPIC cohort). 22](#_Toc46403934)

[**Supplementary Table S10.** Hazard ratios^1^ (95% confidence intervals) for first non-fatal MI or fatal IHD per increment in calibrated intake of plant foods and fibre, stratified by prior disease status (histories of diabetes, hypertension and hyperlipidaemia) (Whole EPIC cohort). 23](#_Toc46403935)

[**Supplementary Table S11.** Associations of fruit, vegetables, legumes, and nuts and seeds with potential mediators in non-cases (Whole EPIC cohort and EPIC-CVD study for biomarker data) 24](#_Toc46403936)

# Supplementary Methods

*Study population*

Data were centralized at the World Health Organization’s International Agency for Research on Cancer (IARC) in Lyon, France. The details of the study design used in the EPIC study have been described elsewhere ^1^.

In the current analyses we included data from the full EPIC cohort and from the EPIC- CVD case-cohort study, which is nested within EPIC and has biomarker data ^13, 14^. For the full EPIC cohort, data were available for 518,502 participants, and those with no dietary data, no non-dietary (lifestyle) data, or those in the top or bottom 1% of the ratio of energy intake to energy requirement, were excluded (n=16,837). We also excluded those who had a self-reported or unknown history of myocardial infarction (MI) or stroke at baseline (n=11,308), 23 cases whose date of diagnosis was after the end of follow-up for each centre, and 23 participants with no follow-up. Finally, a total of 490,311 participants were available for analysis. Data from the EPIC-CVD sub-cohort were used to assess the associations between the exposures and selected biomarkers. The sub-cohort was randomly selected from participants with a stored blood sample, with selection stratified by the 23 EPIC recruitment centres.

*Dietary and lifestyle assessment*

Information on dietary intake during the year before recruitment was collected by country- or center-specific validated dietary questionnaires. Most centers used self-administered food frequency questionnaires, however, in Greece, Ragusa (Italy), Naples (Italy) and Spain participants were interviewed by trained staff members. In Malmö (Sweden), dietary intake was assessed using a modified diet history method which combined information from a self-administered diet questionnaire, a 7-day food registration and a 1-hour interview. With the purpose of improving the comparability of dietary data across the participating centers, dietary intakes from the questionnaires were calibrated using a standardized, computer-based, 24-h dietary recall method in an 8% random sample of the whole EPIC cohort. Information on validation of the dietary questionnaires has been published previously ^2^.

The groupings of fruit and vegetables were based on the food group classification used for EPIC that has been described elsewhere ^3, 4^. In accordance with the popular perception, the grouping for total fruit included fresh fruit only. The subtypes of fruit considered were citrus fruits (e.g. oranges, lemons), apples and pears, and bananas. The grouping for vegetables did not include potatoes or dried beans due to the different carbohydrate and energy composition of these plant foods. The subtypes of vegetables included cruciferous vegetables (e.g., broccoli, cabbage), leafy vegetables (e.g., spinach, lettuce), fruiting vegetables (e.g., tomato, sweet pepper, eggplant), tomatoes (raw, cooked, sauce) and root vegetables (e.g., carrot, beetroot). Not all the centers had data for all subtypes of fruits and vegetables, and this is indicated in all the tables and figures of the manuscript.

Legumes include beans, chickpeas, dried peas and lentils ^5^. For determination of the total amount of nut and seed consumption, we used the value of total intake of nut and seed products, including tree nuts, peanuts, seeds, and nut butter. Because nut intake was assessed in the original FFQs in broad categories (detailed elsewhere ^6^), a stratified analysis by specific types of nuts was not possible. Cereals and cereal products included flour, pasta, rice, other grains, bread, crisp breads, rusks, breakfast cereals, crackers, pastry, and pizza dough.

The dietary fibre variable used in this analysis was obtained using the EPIC Nutrient Data Base (ENDB) in which the dietary fibre composition of foods was standardized to the Association of Analytical Communities (AOAC) method across the participating European countries to take into account the different analytical methods used ^7^. For fruit and vegetables, the AOAC and non-starch polysaccharides (NSP) values (measured using the Englyst method) were assumed to be comparable. Elsewhere, values were standardized as far as possible to AOAC values; for example, for potatoes, legumes, cereals and cakes, AOAC dietary fibre was calculated from NSP values using Mongeau’s regression equation (see Slimani *et al* ^7^ for more detail). The cereal fibre intake variable included fibre from flour, pasta, rice, other grains, bread, crisp breads, rusks, breakfast cereals, crackers, pastry, and pizza dough.

Not all centers had asked questions pertaining to the presence of certain participant characteristics; for example, there was no information on the number of cigarettes smoked (for current smokers) among participants from France or Norway or the Italian centers of Varese, Ragusa, Turin or Naples. There was no information available on a history of angina in the Swedish centers. Missing values were assigned to separate strata for each variable where applicable.

Physical activity was assessed using the Cambridge physical activity index, based on occupational physical activity and cycling/other physical exercise ^8^. At baseline, height and body weight were measured according to standard techniques in most centers, from which body mass index (BMI: <22.5, 22.5-24.9, 25.0-27.4, 27.5-29.9, ≥30.0 kg/m^2^, unknown) was calculated. The exceptions were for two-thirds of women from France, the ‘health-conscious’ participants who were recruited by postal methods and targeted at vegetarians, vegans and other people interested in diet and health in the Oxford (UK) center, and all women from Norway who self-reported their heights and weights.

*Blood samples and blood pressure*

Blood samples for serum were collected from participants according to a standardized protocol at recruitment. Filled syringes were kept at 5–10ºC, protected from light, and transferred to a local laboratory for further processing and aliquoting, except for participants recruited through the Oxford center, where blood samples were collected throughout the United Kingdom and were transported to a laboratory in Norfolk by mail at ambient temperature. Blood fractions (serum, plasma, red cells and buffy coat for DNA extraction) were aliquoted into 0.5 mL straws, which were then heat-sealed at both ends and stored in liquid nitrogen tanks at -196ºC, except in Denmark where samples were stored in 1 ml tubes in nitrogen vapor at −150 °C.

Blood lipids and HbA1c were measured in stored blood samples in the EPIC-CVD study, a case-cohort study nested within the EPIC cohort and designed to investigate the determinants of cardiovascular diseases ^9^. The sub-cohort was randomly selected from participants with a stored blood sample, with selection stratified by the 23 EPIC recruitment centers. Total cholesterol was measured using an enzymatic assay at Stichting Huisartsen Laboratorium (Etten-Leur, Netherlands), and high density lipoprotein (HDL) cholesterol was measured using an enzymatic colorimetric assay method (Roche diagnostics, USA). Non-HDL cholesterol (mmol/L) was calculated by subtracting HDL cholesterol from total cholesterol. Lipid measurements were not available for participants from Norway. HbA1c was measured in the erythrocyte fraction using the Tosoh-G8 HPLC analyser (Tosoh Bioscience, Japan) at Stichting Huisartsen Laboratorium (Etten-Leur, Netherlands).

Blood pressure was measured in most participants at recruitment, except for the centers in Norway, and for Asturias (Spain), or Navarra (Spain). Systolic and diastolic blood pressures were measured by trained personnel; two readings were performed on the right arm in a sitting position (spaced by 1–5 minutes) after an initial resting time of at least 5 minutes by use of a standard mercury manometer or oscillometric device (with the exception of the Danish and Swedish centers where one single measurement was taken in the supine position).

*Ascertainment and verification of cases of ischaemic heart disease*

Validation of suspected events was performed on all ascertained case events (Denmark, Germany, Greece, Italy, and Spain) or on a subset of events (France, Germany, the Netherlands, Sweden, and UK). A range of methods were used to confirm the diagnosis of IHD and included retrieving and assessing medical records or hospital notes, contact with medical professionals, retrieval and assessment of death certificates, or verbal autopsy (next-of-kin).

*Statistical analyses*

All analyses were stratified by sex and EPIC centre and adjusted for age at recruitment (continuous), smoking status (never smoker, former smoker, current smoker <10 cigarettes per day, current smoker 10-19 cigarettes per day, current smoker 20+ cigarettes per day, unknown), history of diabetes (yes, no, unknown), prior hypertension (yes, no , unknown), prior hyperlipidaemia (yes, no , unknown), physical activity (inactive, moderately inactive, moderately active, active, unknown), employment status (employed or student, not employed or student, unknown), level of education completed (none or primary, secondary, vocational or university, unknown), alcohol consumption (non-drinkers and sex-specific fifths of intake among drinkers: cut-points in men were 3.5, 9.7, 18.8 and 36.2 g/d, cut-points in women were 0.9, 2.8, 6.9 and 13.9 g/d), BMI (<22.5, 22.5-24.9, 25.0-27.4, 27.5-29.9, ≥30.0 kg/m^2^, unknown), and observed intakes of total energy, red and processed meat, and cheese (each continuous) intakes, foods that have been previously related to IHD risk in this cohort ^10^.

In further analyses conducted to improve the comparability of dietary data across participating centres and to correct for possible measurement error, the dietary data from the 24-hour recalls were used to provide statistically calibrated estimates of dietary intakes for all individuals in the full cohort, and HRs were calculated for increments (as previously explained) in observed and calibrated intake of each food. Observed food intakes were calibrated using a fixed-effect linear model in which sex and centre specific 24-hour recall data from an 8% random sample of the cohort were regressed on the observed intakes, generating a calibrated intake corresponding to each observed intake, as previously detailed ^11, 12^.

The adjusted model was further mutually adjusted in sensitivity analyses for the other food groups analysed in this study to determine to what extent the associations between the plant foods and IHD risk were independent of the other relevant food groups. We also adjusted the main fully adjusted model for hormone replacement therapy in women. Sensitivity analyses were also performed by repeating the analyses after excluding the first 4 years of follow-up to examine whether the overall results might be influenced by reverse causality. To examine whether associations between plant foods and dietary fibre intake and IHD risk were consistent across sub-groups of other risk factors, we also conducted separate analyses for subsets of sex, smoking status (never, former and current), age at recruitment (<55, 55-64, ≥65 years), BMI (<25.0, 25.0-29.9, ≥30.0 kg/m^2^), and European region (Northern Europe: Denmark, Norway, Sweden; Central Europe: France excepting Provence and SW France, Germany, Netherlands, UK; Southern Europe: Greece, Italy, Spain, Provence, SW France). Tests for heterogeneity of trend between sub-groups were obtained by comparing the risk coefficients for each sub-group using inverse variance weighting, testing for statistical significance using a chi-square test on k-1 degrees of freedom where k is the number of sub-groups.

**References**

1. Riboli E, Hunt KJ, Slimani N, Ferrari P, Norat T, Fahey M, Charrondiere UR, Hemon B, Casagrande C, Vignat J, Overvad K, Tjonneland A, Clavel-Chapelon F, Thiebaut A, Wahrendorf J, Boeing H, Trichopoulos D, Trichopoulou A, Vineis P, Palli D, Bueno-de-Mesquita HB, Peeters PHM, Lund E, Engeset D, Gonzalez CA, Barricarte A, Berglund G, Hallmans G, Day NE, Key TJ, Kaaks R, Saracci R. European prospective investigation into cancer and nutrition (epic): Study populations and data collection. Public Health Nutr. 2002;5:1113-1124

2. Margetts BM, Pietinen P. European prospective investigation into cancer and nutrition: Validity studies on dietary assessment methods. International Journal of Epidemiology. 1997;26:S1-S5

3. Perez-Cornago A, Travis RC, Appleby PN, Tsilidis KK, Tjonneland A, Olsen A, Overvad K, Katzke V, Kuhn T, Trichopoulou A, Peppa E, Kritikou M, Sieri S, Palli D, Sacerdote C, Tumino R, Bueno-de-Mesquita HB, Agudo A, Larranaga N, Molina-Portillo E, Ardanaz E, Chirlaque MD, Lasheras C, Stattin P, Wennberg M, Drake I, Malm J, Schmidt JA, Khaw KT, Gunter M, Freisling H, Huybrechts I, Aune D, Cross AJ, Riboli E, Key TJ. Fruit and vegetable intake and prostate cancer risk in the european prospective investigation into cancer and nutrition (epic). Int J Cancer. 2017;141:287-297

4. Agudo A, Slimani N, Ocke MC, Naska A, Miller AB, Kroke A, Bamia C, Karalis D, Vineis P, Palli D, Bueno-de-Mesquita HB, Peeters PH, Engeset D, Hjartaker A, Navarro C, Martinez Garcia C, Wallstrom P, Zhang JX, Welch AA, Spencer E, Stripp C, Overvad K, Clavel-Chapelon F, Casagrande C, Riboli E. Consumption of vegetables, fruit and other plant foods in the european prospective investigation into cancer and nutrition (epic) cohorts from 10 european countries. Public Health Nutr. 2002;5:1179-1196

5. Slimani N, Fahey M, Welch AA, Wirfalt E, Stripp C, Bergstrom E, Linseisen J, Schulze MB, Bamia C, Chloptsios Y, Veglia F, Panico S, Bueno-de-Mesquita HB, Ocke MC, Brustad M, Lund E, Gonzalez CA, Barcos A, Berglund G, Winkvist A, Mulligan A, Appleby P, Overvad K, Tjonneland A, Clavel-Chapelon F, Kesse E, Ferrari P, Van Staveren WA, Riboli E. Diversity of dietary patterns observed in the european prospective investigation into cancer and nutrition (epic) project. Public Health Nutr. 2002;5:1311-1328

6. Freisling H, Noh H, Slimani N, Chajes V, May AM, Peeters PH, Weiderpass E, Cross AJ, Skeie G, Jenab M, Mancini FR, Boutron-Ruault MC, Fagherazzi G, Katzke VA, Kuhn T, Steffen A, Boeing H, Tjonneland A, Kyro C, Hansen CP, Overvad K, Duell EJ, Redondo-Sanchez D, Amiano P, Navarro C, Barricarte A, Perez-Cornago A, Tsilidis KK, Aune D, Ward H, Trichopoulou A, Naska A, Orfanos P, Masala G, Agnoli C, Berrino F, Tumino R, Sacerdote C, Mattiello A, Bueno-de-Mesquita HB, Ericson U, Sonestedt E, Winkvist A, Braaten T, Romieu I, Sabate J. Nut intake and 5-year changes in body weight and obesity risk in adults: Results from the epic-panacea study. Eur J Nutr. 2018;57:2399-2408

7. Slimani N, Deharveng G, Unwin I, Southgate DAT, Vignat J, Skeie G, Salvini S, Parpinel M, Moller A, Ireland J, Becker W, Farran A, Westenbrink S, Vasilopoulou E, Unwin J, Borgejordet A, Rohrmann S, Church S, Gnagnarella P, Casagrande C, van Bakel M, Niravong M, Boutron-Ruault MC, Stripp C, Tjonneland A, Trichopoulou A, Georga K, Nilsson S, Mattisson I, Ray J, Boeing H, Ocke M, Peters PHM, Jakszyn P, Amiano P, Engeset D, Lund E, de Magistris MS, Sacerdote C, Welch A, Bingham S, Subar AF, Riboli E. The epic nutrient database project (endb): A first attempt to standardize nutrient databases across the 10 european countries participating in the epic study. European Journal of Clinical Nutrition. 2007;61:1037-1056

8. InterAct Consortium, Peters T, Brage S, Westgate K, Franks PW, Gradmark A, Tormo Diaz MJ, Huerta JM, Bendinelli B, Vigl M, Boeing H, Wendel-Vos W, Spijkerman A, Benjaminsen-Borch K, Valanou E, de Lauzon Guillain B, Clavel-Chapelon F, Sharp S, Kerrison N, Langenberg C, Arriola L, Barricarte A, Gonzales C, Grioni S, Kaaks R, Key T, Khaw KT, May A, Nilsson P, Norat T, Overvad K, Palli D, Panico S, Ramon Quiros J, Ricceri F, Sanchez MJ, Slimani N, Tjonneland A, Tumino R, Feskins E, Riboli E, Ekelund U, Wareham N. Validity of a short questionnaire to assess physical activity in 10 european countries. Eur J Epidemiol. 2012;27:15-25

9. Danesh J, Saracci R, Berglund G, Feskens E, Overvad K, Panico S, Thompson S, Fournier A, Clavel-Chapelon F, Canonico M, Kaaks R, Linseisen J, Boeing H, Pischon T, Weikert C, Olsen A, Tjonneland A, Johnsen SP, Jensen MK, Quiros JR, Svatetz CA, Perez MJS, Larranaga N, Sanchez CN, Iribas CM, Bingham S, Khaw KT, Wareham N, Key T, Roddam A, Trichopoulou A, Benetou V, Trichopoulos D, Masala G, Sieri S, Tumino R, Sacerdote C, Mattiello A, Verschuren WMM, Bueno-De-Mesquita HB, Grobbee DE, van der Schouw YT, Melander O, Hallmans G, Wennberg P, Lund E, Kumle M, Skeie G, Ferrari P, Slimani N, Norat T, Riboli E. Epic-heart: The cardiovascular component of a prospective study of nutritional, lifestyle and biological factors in 520,000 middle-aged participants from 10 european countries. Eur J Epidemiol. 2007;22:129-141

10. Key TJ, Appleby PN, Bradbury KE, Sweeting M, Wood A, Johansson I, Kühn T, Steur M, Weiderpass E, Wennberg M, Würtz AML, Agudo A, Andersson J, Arriola L, Boeing H, Boer JMA, Bonnet F, Boutron-Ruault M-C, Cross AJ, Ericson U, Fagherazzi G, Ferrari P, Gunter M, Huerta JM, Katzke V, Khaw K-T, Krogh V, La Vecchia C, Matullo G, Moreno-Iribas C, Naska A, Nilsson LM, Olsen A, Overvad K, Palli D, Panico S, Molina-Portillo E, Quirós JR, Skeie G, Sluijs I, Sonestedt E, Stepien M, Tjønneland A, Trichopoulou A, Tumino R, Tzoulaki I, van der Schouw YT, Verschuren WMM, di Angelantonio E, Langenberg C, Forouhi N, Wareham N, Butterworth A, Riboli E, John D. Meat, fish, dairy products, eggs and risk of ischemic heart disease: A prospective study of 7198 incident cases among 409,885 participants in the pan-european epic cohort. Circulation. 2019;In press

11. Slimani N, Kaaks R, Ferrari P, Casagrande C, Clavel-Chapelon F, Lotze G, Kroke A, Trichopoulos D, Trichopoulou A, Lauria C, Bellegotti M, Ocke MC, Peeters PHM, Engeset D, Lund E, Agudo A, Larranaga N, Mattisson I, Andren C, Johansson I, Davey G, Welch AA, Overvad K, Tjonneland A, van Staveren WA, Saracci R, Riboli E. European prospective investigation into cancer and nutrition (epic) calibration study: Rationale, design and population characteristics. Public Health Nutr. 2002;5:1125-1145

12. Ferrari P, Day NE, Boshuizen HC, Roddam A, Hoffmann K, Thiebaut A, Pera G, Overvad K, Lund E, Trichopoulou A, Tumino R, Gullberg B, Norat T, Slimani N, Kaaks R, Riboli E. The evaluation of the diet/disease relation in the epic study: Considerations for the calibration and the disease models. Int J Epidemiol. 2008;37:368-378

# Supplementary Tables

| **Supplementary Table S1.** Observed intakes of foods and fibre in each EPIC country. | | | | | | | | | | |
| --- | --- | --- | --- | --- | --- | --- | --- | --- | --- | --- |
| **Foods, g/day, medians (IQR)** | Denmark | France | Germany | Greece | Italy | Norway | Spain | Sweden | The Netherlands | UK |
| Fruit and vegetables | 357 (203) | 543 (238) | 263 (129) | 818 (322) | 514 (251) | 299 (164) | 568 (295) | 300 (185) | 328 (156) | 543 (238) |
| Vegetables | 178 (100) | 284 (136) | 124 (60) | 458 (193) | 175 (98) | 142 (80) | 250 (148) | 124 (95) | 131 (52) | 284 (136) |
| Fruiting vegetables^1^ | 35 (25) | 69 (57) | 56 (35) | 282 (125) | 81 (53) |  | 105 (76) | 50 (50) | 42 (23) | 69 (57) |
| Leafy vegetables^2^ | 2 (2) | 72 (43) | 11 (10) | 61 (44) | 35 (27) |  | 69 (63) | 16 (19) | 26 (17) | 72 (43) |
| Cruciferous vegetables^3^ | 15 (12) | 22 (23) | 16 (14) | 31 (25) | 7 (8) | 46 (39) | 9 (21) | 13 (22) | 24 (18) | 22 (23) |
| Root vegetables | 29 (42) | 29 (25) | 14 (13) | 31 (33) | 16 (21) | 45 (33) | 8 (14) | 29 (36) | 19 (14) | 29 (25) |
| Fruit | 179 (147) | 259 (167) | 139 (95) | 361 (195) | 339 (209) | 157 (121) | 317 (229) | 176 (128) | 197 (137) | 259 (167) |
| Citrus fruit | 35 (47) | 38 (41) | 20 (19) | 96 (88) | 85 (81) | 26 (37) | 99 (104) | 42 (49) | 47 (41) | 38 (41) |
| Apples & pears | 91 (96) | 34 (36) | 37 (40) | 67 (56) | 120 (100) | 60 (56) | 96 (134) | 44 (56) | 63 (49) | 34 (36) |
| Banana | 25.28 (36.86) | 6.75 (9.69) | 17.55 (21.19) | 10.07 (17.55) | 14.87 (25.97) | 41.63 (45.40) | 5.68 (18.92) | 42.51 (44.69) | 24.78 (24.31) | 6.75 (9.69) |
| Legumes^4^ |  | 18.77 (21.84) | 4.46 (6.24) | 21.71 (15.82) | 12.45 (19.43) |  | 51.58 (37.76) | 3.83 (10.46) | 9.32 (10.07) | 18.77 (21.84) |
| Nuts and seeds^3^ | 1.8 (4.0) | 5.5 (8.4) | 3.5 (7.4) | 5.9 (8.3) | 0.9 (2.0) | 2.2 (3.6) | 4.3 (11.0) | 1.6 (4.7) | 8.8 (12.6) | 5.5 (8.4) |
| Cereals and cereal products | 212 (82) | 209 (103) | 195 (79) | 208 (82) | 329 (155) | 196 (66) | 208 (99) | 208 (112) | 196 (91) | 209 (103) |
| Total dietary fibre | 25.02 (8.19) | 22.62 (6.97) | 21.60 (6.85) | 21.87 (6.71) | 22.31 (7.62) | 20.57 (6.03) | 24.55 (8.32) | 19.81 (7.11) | 23.00 (6.50) | 22.62 (6.97) |
| Fruit and vegetable fibre | 7.9 (4.4) | 11.4 (5.0) | 5.8 (2.8) | 12.2 (4.8) | 10.8 (5.3) | 6.9 (3.7) | 10.6 (5.5) | 6.0 (3.8) | 7.6 (3.3) | 11.4 (5.0) |
| Vegetable fibre | 4.3 (2.4) | 6.4 (3.1) | 3.0 (1.5) | 8.1 (3.4) | 4.1 (2.3) | 3.7 (2.2) | 5.2 (3.3) | 2.4 (1.8) | 3.8 (1.5) | 6.4 (3.1) |
| Fruit fibre | 3.6 (3.0) | 5.0 (3.3) | 2.8 (1.9) | 4.1 (2.5) | 6.7 (4.3) | 3.2 (2.5) | 5.3 (4.0) | 3.6 (2.8) | 3.8 (2.7) | 5.0 (3.3) |
| Cereal fibre | 13.6 (5.8) | 6.1 (3.4) | 9.8 (4.8) | 6.5 (3.1) | 7.7 (3.8) | 10.3 (3.9) | 6.1 (3.6) | 9.5 (4.8) | 8.2 (3.9) | 6.1 (3.4) |
| Abbreviations: IQR, interquartile range; UK, United Kingdom.  ^1^ Such as tomatoes, cucumbers and sweet peppers; unavailable for Norway.  ^2^ Unavailable for Norway and Umea.  ^3^ Unavailable for Umea.  ^4^ Unavailable for Denmark and Norway. | | | | | | | | | | |

| **Supplementary Table S2.** Hazard ratios^1^ (95% confidence intervals) for any first fatal IHD or non-fatal MI by overall fifths and increments of observed intake in plant foods and dietary fibre (Whole EPIC cohort). | | | | | | | | | |
| --- | --- | --- | --- | --- | --- | --- | --- | --- | --- |
|  | **Fifths of observed intake** | | | | |  | **Per increment** | | |
|  | **1** | **2** | **3** | **4** | **5** |  | **Increment** | **Observed intake** | **P-trend** |
| **Fruit and vegetables, g/day** | ≤ 223.2 | >223.2 to ≤ 333.4 | >333.4 to ≤ 455.5 | >455.5 to ≤ 630.6 | > 630.6 |  | 200 |  |  |
| Cases, *n* | 2473 | 1924 | 1677 | 1304 | 1126 |  |  | 8504 |  |
| HR (95% CI) | 1 ref | 0.87 (0.81 - 0.92) | 0.84 (0.79 - 0.90) | 0.77 (0.72 - 0.83) | 0.76 (0.70 - 0.82) |  |  | 0.92 (0.90 - 0.94) | <0.001 |
| Adjusted HR (95% CI) ^2^ | 1 ref | 0.97 (0.91 - 1.03) | 0.98 (0.92 - 1.05) | 0.92 (0.85 - 0.99) | 0.92 (0.84 - 1.00) |  |  | 0.97 (0.95 - 0.99) | 0.013 |
| **Vegetables, g/day** | ≤ 98.2 | >98.2 to ≤ 147.1 | >147.1 to ≤ 209.0 | >209.0 to ≤ 307.3 | > 307.3 |  | 100 |  |  |
| Cases, *n* | 2430 | 1873 | 1714 | 1351 | 1136 |  |  | 8504 |  |
| HR (95% CI) | 1 ref | 0.92 (0.86 - 0.98) | 0.88 (0.83 - 0.94) | 0.79 (0.74 - 0.85) | 0.81 (0.75 - 0.88) |  |  | 0.95 (0.93 - 0.97) | <0.001 |
| Adjusted HR (95% CI) ^2^ | 1 ref | 1.01 (0.95 - 1.07) | 1.00 (0.94 - 1.07) | 0.93 (0.87 - 1.01) | 0.96 (0.88 - 1.04) |  |  | 0.99 (0.97 - 1.01) | 0.204 |
| **Fruiting vegetables^3^, g/day** | ≤ 24.1 | >24.1 to ≤ 42.8 | >42.8 to ≤ 66.1 | >66.1 to ≤ 105.4 | > 105.4 |  | 50 |  |  |
| Cases, *n* | 2440 | 2041 | 1551 | 1290 | 1144 |  |  | 8466 |  |
| HR (95% CI) | 1 ref | 0.89 (0.83 - 0.94) | 0.82 (0.77 - 0.87) | 0.82 (0.76 - 0.88) | 0.80 (0.74 - 0.87) |  |  | 0.95 (0.93 - 0.97) | <0.001 |
| Adjusted HR (95% CI) ^2^ | 1 ref | 0.96 (0.90 - 1.02) | 0.91 (0.85 - 0.98) | 0.93 (0.87 - 1.01) | 0.92 (0.84 - 1.00) |  |  | 0.98 (0.96 - 1.01) | 0.187 |
| **Leafy vegetables^4^, g/day** | ≤ 3.5 | >3.5 to ≤ 11.6 | >11.6 to ≤ 25.7 | >25.7 to ≤ 56.5 | > 56.5 |  | 50 |  |  |
| Cases, *n* | 3094 | 1661 | 1320 | 1082 | 685 |  |  | 7842 |  |
| HR (95% CI) | 1 ref | 0.84 (0.79 - 0.90) | 0.84 (0.77 - 0.90) | 0.85 (0.78 - 0.92) | 0.82 (0.73 - 0.92) |  |  | 0.96 (0.91 - 1.00) | 0.055 |
| Adjusted HR (95% CI) ^2^ | 1 ref | 0.96 (0.89 - 1.02) | 1.00 (0.93 - 1.09) | 1.01 (0.92 - 1.10) | 0.96 (0.85 - 1.08) |  |  | 0.99 (0.94 - 1.03) | 0.511 |
| **Cruciferous vegetables^5^, g/day** | ≤ 3.4 | >3.4 to ≤ 11.7 | >11.7 to ≤ 23.1 | >23.1 to ≤ 43.7 | > 43.7 |  | 50 |  |  |
| Cases, *n* | 2010 | 1741 | 1463 | 1272 | 1394 |  |  | 7880 |  |
| HR (95% CI) | 1 ref | 0.96 (0.89 - 1.04) | 0.89 (0.82 - 0.96) | 0.93 (0.86 - 1.01) | 0.95 (0.87 - 1.04) |  |  | 1.01 (0.98 - 1.05) | 0.449 |
| Adjusted HR (95% CI) ^2^ | 1 ref | 1.00 (0.93 - 1.08) | 0.97 (0.90 - 1.05) | 1.01 (0.93 - 1.09) | 1.03 (0.93 - 1.12) |  |  | 1.02 (0.99 - 1.06) | 0.250 |
| **Root vegetables, g/day** | ≤ 4.9 | >4.9 to ≤ 12.5 | >12.5 to ≤ 24.8 | >24.8 to ≤ 44.2 | > 44.2 |  | 50 |  |  |
| Cases, *n* | 2103 | 1950 | 1622 | 1381 | 1448 |  |  | 8504 |  |
| HR (95% CI) | 1 ref | 0.92 (0.87 - 0.98) | 0.87 (0.82 - 0.94) | 0.83 (0.77 - 0.89) | 0.80 (0.75 - 0.86) |  |  | 0.91 (0.88 - 0.95) | <0.001 |
| Adjusted HR (95% CI) ^2^ | 1 ref | 1.00 (0.93 - 1.06) | 0.98 (0.92 - 1.05) | 0.95 (0.88 - 1.02) | 0.94 (0.87 - 1.01) |  |  | 0.96 (0.93 - 1.00) | 0.059 |
| **Fruit, g/day** | ≤ 91.7 | >91.7 to ≤ 156.8 | >156.8 to ≤ 238.0 | >238.0 to ≤ 346.8 | > 346.8 |  | 100 |  |  |
| Cases, *n* | 2336 | 1951 | 1593 | 1345 | 1279 |  |  | 8504 |  |
| HR (95% CI) | 1 ref | 0.91 (0.86 - 0.97) | 0.81 (0.76 - 0.87) | 0.78 (0.73 - 0.84) | 0.79 (0.73 - 0.85) |  |  | 0.95 (0.94 - 0.96) | <0.001 |
| Adjusted HR (95% CI) ^2^ | 1 ref | 1.00 (0.94 - 1.06) | 0.93 (0.87 - 0.99) | 0.91 (0.85 - 0.98) | 0.93 (0.86 - 1.01) |  |  | 0.98 (0.97 - 1.00) | 0.015 |
| **Citrus fruit, g/day** | ≤ 6.8 | >6.8 to ≤ 17.4 | >17.4 to ≤ 42.8 | >42.8 to ≤ 82.9 | > 82.9 |  | 50 |  |  |
| Cases, *n* | 2140 | 1912 | 1612 | 1494 | 1346 |  |  | 8504 |  |
| HR (95% CI) | 1 ref | 0.88 (0.83 - 0.94) | 0.85 (0.80 - 0.91) | 0.84 (0.79 - 0.90) | 0.79 (0.73 - 0.85) |  |  | 0.96 (0.94 - 0.98) | <0.001 |
| Adjusted HR (95% CI) ^2^ | 1 ref | 0.96 (0.90 - 1.02) | 0.95 (0.89 - 1.02) | 0.96 (0.90 - 1.03) | 0.90 (0.84 - 0.97) |  |  | 0.98 (0.96 - 1.00) | 0.109 |
| **Apples & pears, g/day** | ≤ 10.3 | >10.3 to ≤ 31.0 | >31.0 to ≤ 62.0 | >62.0 to ≤ 110.0 | > 110.0 |  | 50 |  |  |
| Cases, *n* | 1733 | 1763 | 1424 | 1669 | 1915 |  |  | 8504 |  |
| HR (95% CI) | 1 ref | 0.85 (0.80 - 0.91) | 0.81 (0.75 - 0.87) | 0.79 (0.74 - 0.85) | 0.78 (0.73 - 0.84) |  |  | 0.97 (0.96 - 0.99) | <0.001 |
| Adjusted HR (95% CI) ^2^ | 1 ref | 0.93 (0.87 - 0.99) | 0.93 (0.86 - 1.00) | 0.92 (0.85 - 0.99) | 0.91 (0.85 - 0.98) |  |  | 0.99 (0.98 - 1.00) | 0.164 |
|  |  |  |  |  |  |  |  |  |  |
| **Banana^6^, g/day** | ≤ 0.0 | >0.012 to ≤ 7.1 | >7.1 to ≤ 15.7 | >15.7 to ≤ 43.0 | > 43.0 |  | 50 |  |  |
| Cases, *n* | 1645 | 1583 | 1696 | 1736 | 1844 |  |  | 8504 |  |
| HR (95% CI) | 1 ref | 0.95 (0.88 - 1.03) | 0.90 (0.84 - 0.98) | 0.84 (0.77 - 0.90) | 0.80 (0.75 - 0.87) |  |  | 0.92 (0.90 - 0.95) | <0.001 |
| Adjusted HR (95% CI) ^2^ | 1 ref | 1.00 (0.92 - 1.08) | 0.99 (0.91 - 1.06) | 0.95 (0.88 - 1.02) | 0.94 (0.87 - 1.01) |  |  | 0.97 (0.94 - 1.00) | 0.060 |
| **Legumes^7^, g/day** | ≤ 0.7 | >0.7 to ≤ 5.8 | >5.8 to ≤ 12.3 | >12.3 to ≤ 30.7 | > 30.7 |  | 20 |  |  |
| Cases, *n* | 2293 | 1074 | 1160 | 874 | 1131 |  |  | 6532 |  |
| HR (95% CI) | 1 ref | 1.01 (0.92 - 1.11) | 1.01 (0.93 - 1.10) | 1.02 (0.92 - 1.12) | 1.02 (0.92 - 1.12) |  |  | 1.00 (0.98 - 1.03) | 0.788 |
| Adjusted HR (95% CI) ^2^ | 1 ref | 1.02 (0.93 - 1.12) | 1.00 (0.92 - 1.09) | 0.99 (0.90 - 1.09) | 1.00 (0.91 - 1.10) |  |  | 1.00 (0.97 - 1.02) | 0.843 |
| **Nuts and seeds^5,6^, g/day** | ≤ 0.0 | >0.006 to ≤ 0.5 | >0.5 to ≤ 2.0 | >2.0 to ≤ 5.3 | > 5.3 |  | 10 |  |  |
| Cases, *n* | 3296 | 1234 | 1830 | 1158 | 986 |  |  | 7880 |  |
| HR (95% CI) | 1 ref | 0.91 (0.83 - 0.99) | 0.85 (0.79 - 0.90) | 0.81 (0.75 - 0.87) | 0.76 (0.70 - 0.82) |  |  | 0.92 (0.89 - 0.95) | <0.001 |
| Adjusted HR (95% CI) ^2^ | 1 ref | 0.99 (0.90 - 1.08) | 0.97 (0.91 - 1.04) | 0.96 (0.89 - 1.03) | 0.93 (0.86 - 1.01) |  |  | 0.97 (0.94 - 1.00) | 0.068 |
| **Cereals and cereal products** | ≤ 131.2 | >131.2 to ≤ 176.5 | >176.5 to ≤ 223.8 | >223.8 to ≤ 290.5 | > 290.5 |  | 200 |  |  |
| Cases, *n* | 1945 | 1632 | 1558 | 1586 | 1783 |  |  | 8504 |  |
| HR (95% CI) | 1 ref | 0.90 (0.84 - 0.96) | 0.87 (0.81 - 0.93) | 0.85 (0.79 - 0.91) | 0.85 (0.80 - 0.91) |  |  | 0.92 (0.88 - 0.96) | <0.001 |
| Adjusted HR (95% CI) ^2^ | 1 ref | 0.97 (0.91 - 1.04) | 0.96 (0.89 - 1.03) | 0.98 (0.91 - 1.06) | 1.01 (0.94 - 1.10) |  |  | 1.02 (0.97 - 1.07) | 0.490 |
|  |  |  |  |  |  |  |  |  |  |
| **Total dietary fibre, g/day** | ≤ 16.4 | >16.4 to ≤ 20.1 | >20.1 to ≤ 23.6 | >23.6 to ≤ 28.5 | > 28.5 |  | 10 |  |  |
| Cases, *n* | 1982 | 1646 | 1518 | 1603 | 1755 |  |  | 8504 |  |
| HR (95% CI) | 1 ref | 0.87 (0.81 - 0.93) | 0.79 (0.74 - 0.85) | 0.79 (0.74 - 0.85) | 0.75 (0.70 - 0.81) |  |  | 0.88 (0.86 - 0.91) | <0.001 |
| Adjusted HR (95% CI) ^2^ | 1 ref | 0.94 (0.88 - 1.00) | 0.89 (0.83 - 0.95) | 0.91 (0.85 - 0.98) | 0.91 (0.83 - 0.99) |  |  | 0.96 (0.92 - 0.99) | 0.019 |
| **Fruit and vegetable fibre, g/day** | ≤ 4.8 | >4.8 to ≤ 6.9 | >6.9 to ≤ 9.2 | >9.2 to ≤ 12.4 | > 12.4 |  | 4 |  |  |
| Cases, *n* | 2546 | 1881 | 1576 | 1320 | 1181 |  |  | 8504 |  |
| HR (95% CI) | 1 ref | 0.86 (0.81 - 0.92) | 0.81 (0.76 - 0.86) | 0.77 (0.72 - 0.83) | 0.79 (0.73 - 0.85) |  |  | 0.93 (0.91 - 0.95) | <0.001 |
| Adjusted HR (95% CI) ^2^ | 1 ref | 0.96 (0.90 - 1.02) | 0.93 (0.87 - 0.99) | 0.91 (0.84 - 0.98) | 0.94 (0.87 - 1.02) |  |  | 0.98 (0.95 - 1.00) | 0.033 |
| **Vegetable fibre, g/day** | ≤ 2.3 | >2.3 to ≤ 3.4 | >3.4 to ≤ 4.7 | >4.7 to ≤ 6.7 | > 6.7 |  | 2 |  |  |
| Cases, *n* | 2555 | 1808 | 1632 | 1347 | 1162 |  |  | 8504 |  |
| HR (95% CI) | 1 ref | 0.91 (0.85 - 0.96) | 0.90 (0.84 - 0.96) | 0.84 (0.78 - 0.90) | 0.83 (0.77 - 0.90) |  |  | 0.96 (0.94 - 0.98) | <0.001 |
| Adjusted HR (95% CI) ^2^ | 1 ref | 0.99 (0.93 - 1.06) | 1.01 (0.95 - 1.08) | 0.96 (0.89 - 1.03) | 0.96 (0.88 - 1.05) |  |  | 0.99 (0.97 - 1.01) | 0.297 |
| **Fruit fibre, g/day** | ≤ 1.7 | >1.7 to ≤ 2.8 | >2.8 to ≤ 4.2 | >4.2 to ≤ 6.2 | > 6.2 |  | 2 |  |  |
| Cases, *n* | 2311 | 1842 | 1617 | 1419 | 1315 |  |  | 8504 |  |
| HR (95% CI) | 1 ref | 0.88 (0.83 - 0.94) | 0.82 (0.77 - 0.88) | 0.79 (0.74 - 0.85) | 0.77 (0.72 - 0.83) |  |  | 0.95 (0.93 - 0.96) | <0.001 |
| Adjusted HR (95% CI) ^2^ | 1 ref | 0.97 (0.91 - 1.03) | 0.94 (0.88 - 1.00) | 0.93 (0.87 - 1.00) | 0.91 (0.84 - 0.98) |  |  | 0.98 (0.97 - 1.00) | 0.032 |
| **Cereal fibre, g/day** | ≤ 4.6 | >4.6 to ≤ 6.7 | >6.7 to ≤ 8.9 | >8.9 to ≤ 12.2 | > 12.2 |  | 4 |  |  |
| Cases, *n* | 1399 | 1508 | 1575 | 1795 | 2227 |  |  | 8504 |  |
| HR (95% CI) | 1 ref | 0.92 (0.85 - 0.99) | 0.86 (0.80 - 0.93) | 0.83 (0.77 - 0.89) | 0.77 (0.71 - 0.83) |  |  | 0.94 (0.93 - 0.96) | <0.001 |
| Adjusted HR (95% CI) ^2^ | 1 ref | 0.98 (0.91 - 1.05) | 0.95 (0.88 - 1.03) | 0.94 (0.87 - 1.01) | 0.92 (0.84 - 1.00) |  |  | 0.99 (0.97 - 1.01) | 0.405 |
| ^1^ Cox regression analysis. All models are stratified by sex and EPIC centre. | | | | | | | | | |
| ^2^ Additionally adjusted for age (continuous), smoking status and number of cigarettes per day (never smoker, former smoker, current smoker <10 cigs/d, current smoker 10-19 cigs/d, current smoker 20+ cigs/d, unknown), histories of diabetes, hypertension and hyperlipidaemia (each yes, no, unknown), Cambridge physical activity index (inactive, moderately inactive, moderately active, active, unknown), employment status (employed or student, not employed or student, unknown), level of education completed (none or primary, secondary, vocational or university, unknown), current alcohol consumption (non-drinkers and sex-specific fifths of intake among drinkers), BMI (<22.5, 22.5-24.9, 25.0-27.4, 27.5-29.9, ≥30.0 kg/m^2^, unknown), and observed intakes of total energy, red and processed meat, and cheese (each continuous), and stratified by sex and EPIC centre | | | | | | | | | |
| ^3^ Such as tomatoes, cucumbers and sweet peppers; unavailable for Norway.  ^4^ Unavailable for Norway and Umea.  ^5^ Unavailable for Umea.  ^6^ non-consumers vs. Q1 to Q4 of intakes in consumers, due to a higher number of non-consumers (n=102,742 for bananas and n=121,683 for nuts and seeds)  ^7^ Unavailable for Denmark and Norway.  Abbreviations: Body mass index (BMI), ischaemic heart disease (IHD), myocardial infarction (MI). | | | | | | | | | |

| **Supplementary Table S3**. Hazard ratios^1^ (95% confidence intervals) for any first fatal IHD or non-fatal MI per increment in calibrated intake of fruit, vegetables and fibre, after further mutual adjustment of significant foods or hormone replacement therapy (Whole EPIC cohort). | | | | | | |
| --- | --- | --- | --- | --- | --- | --- |
| Food | **Increment (g/day)** | **Mutually plant foods adjustment** | **P for trend^2^** |  | **Hormone replacement therapy adjustment** | **P for trend^2^** |
| Fruit and vegetables | 200 | 0.94 (0.90 - 0.99) | 0.009 |  | 0.94 (0.90 - 0.99) | 0.008 |
| Vegetables | 100 | 0.97 (0.91 - 1.03) | 0.321 |  | 0.95 (0.90 - 1.02) | 0.142 |
| Fruiting vegetables^3^ | 50 | 1.11 (0.98 - 1.26) | 0.113 |  | 0.95 (0.90 - 1.01) | 0.087 |
| Leafy vegetables^4^ | 50 | 0.99 (0.86 - 1.14) | 0.875 |  | 0.97 (0.85 - 1.12) | 0.705 |
| Cruciferous vegetables^5^ | 50 | 0.96 (0.91 - 1.02) | 0.180 |  | 1.09 (0.96 - 1.24) | 0.189 |
| Root vegetables | 50 | 0.93 (0.85 - 1.02) | 0.141 |  | 0.91 (0.83 - 1.00) | 0.058 |
| Fruit | 100 | 0.97 (0.95 - 1.00) | 0.035 |  | 0.97 (0.95 - 1.00) | 0.021 |
| Citrus fruit | 50 | 0.99 (0.95 - 1.02) | 0.378 |  | 0.98 (0.95 - 1.02) | 0.304 |
| Apples & pears | 50 | 0.99 (0.97 - 1.01) | 0.263 |  | 0.99 (0.97 - 1.01) | 0.187 |
| Banana | 50 | 0.92 (0.86 - 0.98) | 0.008 |  | 0.92 (0.86 - 0.97) | 0.006 |
| Legumes^6^ | 20 | 1.01 (0.97 - 1.06) | 0.631 |  | 1.01 (0.96 - 1.06) | 0.694 |
| Nuts and seeds^5^ | 10 | 0.91 (0.83 - 0.99) | 0.030 |  | 0.90 (0.82 - 0.98) | 0.020 |
| Cereals and cereal products | 200 | 1.03 (0.92 - 1.16) | 0.601 |  | 1.04 (0.92 - 1.16) | 0.561 |
|  |  |  |  |  |  |  |
| Total dietary fibre | 10 | 0.90 (0.83 - 0.96) | 0.002 |  | 0.91 (0.85 - 0.98) | 0.014 |
| Fruit and vegetable fibre | 4 | 0.94 (0.90 - 0.99) | 0.009 |  | 0.95 (0.91 - 0.99) | 0.021 |
| Vegetable fibre | 2 | 0.96 (0.90 - 1.02) | 0.162 |  | 0.96 (0.90 - 1.02) | 0.188 |
| Fruit fibre | 2 | 0.97 (0.94 - 0.99) | 0.016 |  | 0.97 (0.95 - 1.00) | 0.045 |
| Cereal fibre | 4 | 0.96 (0.92 - 1.00) | 0.053 |  | 0.98 (0.94 - 1.02) | 0.282 |
| ^1^ Hazard ratios are adjusted for age (continuous), smoking status and number of cigarettes per day (never smoker, former smoker, current smoker <10 cigs/d, current smoker 10-19 cigs/d, current smoker 20+ cigs/d, unknown), histories of diabetes, hypertension and hyperlipidaemia (each yes, no, unknown), Cambridge physical activity index (inactive, moderately inactive, moderately active, active, unknown), employment status (employed or student, not employed or student, unknown), level of education completed (none or primary, secondary, vocational or university, unknown), current alcohol consumption (non-drinkers and sex-specific fifths of intake among drinkers), BMI (<22.5, 22.5-24.9, 25.0-27.4, 27.5-29.9, ≥30.0 kg/m^2^, unknown), and observed intakes of total energy, and stratified by sex and EPIC centre.  The mutually plant foods adjustment model included the model above plus mutual adjustment of the other plant foods. The model adjusted by hormonal therapy included the model above plus hormone replacement therapy.  ^2^ Tests of trend were performed using the calibrated intake (continuous).  ^3^ Such as tomatoes, cucumbers and sweet peppers; unavailable for Norway.  ^4^ Unavailable for Norway and Umea.  ^5^ Unavailable for Umea.  ^6^ Unavailable for Denmark and Norway.  Abbreviations: Body mass index (BMI), ischaemic heart disease (IHD), myocardial infarction (MI). | | | | | | |

| **Supplementary Table S4**. Hazard ratios^1^ (95% confidence intervals) for any first fatal IHD or non-fatal MI per increment in calibrated intake of fruit, vegetables and fibre excluding the first 4 years of follow-up (Whole EPIC cohort). | | | | |
| --- | --- | --- | --- | --- |
| **Food** | **No. of cases** | **Increment (g/day)** | **Calibrated intake, HR (95% CI)** | **P trend^2^** |
| Fruit and vegetables | 6463 | 200 | 0.95 (0.91-1.00) | 0.053 |
| Vegetables | 6463 | 100 | 0.96 (0.89-1.03) | 0.20 |
| Fruiting vegetables^3^ | 6449 | 50 | 0.95 (0.89-1.01) | 0.094 |
| Leafy vegetables^4^ | 5968 | 50 | 0.97 (0.82-1.13) | 0.68 |
| Cruciferous vegetables^5^ | 5982 | 50 | 1.09 (0.94-1.26) | 0.27 |
| Root vegetables | 6463 | 50 | 0.92 (0.83-1.02) | 0.11 |
| Fruit | 6463 | 100 | 0.98 (0.95-1.01) | 0.13 |
| Citrus fruit | 6463 | 50 | 0.98 (0.95-1.02) | 0.34 |
| Apples & pears | 6463 | 50 | 0.99 (0.97-1.02) | 0.67 |
| Banana | 6463 | 50 | 0.93 (0.87-1.00) | 0.047 |
| Legumes^6^ | 4962 | 20 | 1.00 (0.95-1.06) | 0.88 |
| Nuts and seeds^5^ | 5982 | 10 | 0.90 (0.82-1.00) | 0.049 |
| Cereals and cereal products | 6463 | 200 | 1.01 (0.88-1.15) | 0.91 |
|  |  |  |  |  |
| Total dietary fibre | 6463 | 10 | 0.92 (0.85-1.00) | 0.058 |
| Fruit and vegetable fibre | 6463 | 4 | 0.96 (0.91-1.01) | 0.10 |
| Vegetable fibre | 6463 | 2 | 0.96 (0.89-1.03) | 0.23 |
| Fruit fibre | 6463 | 2 | 0.98 (0.95-1.01) | 0.24 |
| Cereal fibre | 6463 | 4 | 0.97 (0.93-1.02) | 0.29 |
| ^1^ Hazard ratios are adjusted for age (continuous), smoking status and number of cigarettes per day (never smoker, former smoker, current smoker <10 cigs/d, current smoker 10-19 cigs/d, current smoker 20+ cigs/d, unknown), histories of diabetes, hypertension and hyperlipidaemia (each yes, no, unknown), Cambridge physical activity index (inactive, moderately inactive, moderately active, active, unknown), employment status (employed or student, not employed or student, unknown), level of education completed (none or primary, secondary, vocational or university, unknown), current alcohol consumption (non-drinkers and sex-specific fifths of intake among drinkers), BMI (<22.5, 22.5-24.9, 25.0-27.4, 27.5-29.9, ≥30.0 kg/m^2^, unknown), and calibrated intakes of total energy, red and processed meat, and cheese (each continuous), and stratified by sex and EPIC centre.  ^2^ Tests of trend were performed using the calibrated intake.  ^3^ Such as tomatoes, cucumbers and sweet peppers; unavailable for Norway.  ^4^ Unavailable for Norway and Umea.  ^5^ Unavailable for Umea.  ^6^ Unavailable for Denmark and Norway.  Abbreviations: Body mass index (BMI), ischaemic heart disease (IHD), myocardial infarction (MI). | | | | |

| **Supplementary Table S5**. Hazard ratios^1^ (95% confidence intervals) for first non-fatal MI or fatal IHD per increment in calibrated intake of plant foods and fibre subdivided by smoking status (Whole EPIC cohort). | | | | | | | | | | | |
| --- | --- | --- | --- | --- | --- | --- | --- | --- | --- | --- | --- |
| **Food** | **Increment (g/day)** |  | **Never smoker (2457 cases)** | |  | **Former smoker (2559 cases)** | |  | **Current smoker (3409 cases)** | | **P for heterogeneity^7^** |
|  |  |  | **HR (95% CI)** | **P trend^2^** |  | **HR (95% CI)** | **P trend^2^** |  | **HR (95% CI)** | **P trend^2^** |  |
| Fruit and vegetables | 200 |  | 0.96 (0.89-1.05) | 0.37 |  | 0.94 (0.87-1.02) | 0.15 |  | 0.93 (0.86-0.99) | 0.029 | 0.77 |
| Vegetables | 100 |  | 0.99 (0.88-1.11) | 0.84 |  | 0.96 (0.85-1.07) | 0.43 |  | 0.91 (0.83-1.01) | 0.077 | 0.61 |
| Fruiting vegetables^3^ | 50 |  | 1.01 (0.91-1.12) | 0.86 |  | 0.93 (0.84-1.03) | 0.19 |  | 0.92 (0.84-1.00) | 0.058 | 0.37 |
| Leafy vegetables^4^ | 50 |  | 0.95 (0.72-1.25) | 0.71 |  | 0.86 (0.66-1.13) | 0.28 |  | 1.05 (0.86-1.29) | 0.64 | 0.51 |
| Cruciferous vegetables^5^ | 50 |  | 1.09 (0.85-1.40) | 0.49 |  | 1.12 (0.91-1.37) | 0.30 |  | 1.04 (0.83-1.31) | 0.70 | 0.91 |
| Root vegetables | 50 |  | 0.95 (0.80-1.12) | 0.54 |  | 0.95 (0.81-1.11) | 0.50 |  | 0.84 (0.71-0.98) | 0.030 | 0.46 |
| Fruit | 100 |  | 0.98 (0.93-1.03) | 0.42 |  | 0.97 (0.93-1.01) | 0.17 |  | 0.96 (0.93-1.00) | 0.076 | 0.88 |
| Citrus fruit | 50 |  | 1.02 (0.95-1.08) | 0.63 |  | 0.93 (0.88-0.99) | 0.030 |  | 1.00 (0.95-1.05) | 0.98 | 0.13 |
| Apples & pears | 50 |  | 1.01 (0.97-1.05) | 0.67 |  | 0.97 (0.94-1.01) | 0.18 |  | 0.98 (0.94-1.01) | 0.16 | 0.37 |
| Banana | 50 |  | 0.91 (0.81-1.02) | 0.099 |  | 0.97 (0.88-1.08) | 0.59 |  | 0.88 (0.79-0.98) | 0.017 | 0.39 |
| Legumes^6^ | 20 |  | 1.11 (1.02-1.21) | 0.019 |  | 0.98 (0.90-1.06) | 0.59 |  | 0.99 (0.92-1.06) | 0.70 | 0.066 |
| Nuts and seeds^5^ | 10 |  | 1.03 (0.87-1.21) | 0.75 |  | 0.88 (0.75-1.03) | 0.10 |  | 0.85 (0.74-0.98) | 0.024 | 0.21 |
| Cereals and cereal products | 200 |  | 0.96 (0.75-1.21) | 0.71 |  | 1.00 (0.81-1.22) | 0.98 |  | 1.09 (0.91-1.31) | 0.34 | 0.65 |
|  |  |  |  |  |  |  |  |  |  |  |  |
| Total dietary fibre | 10 |  | 1.05 (0.91-1.22) | 0.47 |  | 0.85 (0.75-0.97) | 0.016 |  | 0.88 (0.79-0.98) | 0.025 | 0.066 |
| Fruit and vegetable fibre | 4 |  | 0.98 (0.90-1.06) | 0.61 |  | 0.94 (0.87-1.02) | 0.16 |  | 0.93 (0.87-1.00) | 0.041 | 0.65 |
| Vegetable fibre | 2 |  | 1.00 (0.88-1.12) | 0.93 |  | 0.95 (0.85-1.07) | 0.42 |  | 0.92 (0.83-1.02) | 0.099 | 0.60 |
| Fruit fibre | 2 |  | 0.99 (0.94-1.04) | 0.65 |  | 0.96 (0.92-1.01) | 0.12 |  | 0.97 (0.93-1.01) | 0.14 | 0.76 |
| Cereal fibre | 4 |  | 1.05 (0.96-1.14) | 0.27 |  | 0.91 (0.84-0.98) | 0.016 |  | 0.99 (0.92-1.05) | 0.67 | 0.050 |
| ^1^ Hazard ratios are adjusted for age (continuous), smoking status and number of cigarettes per day (never smoker, former smoker, current smoker <10 cigs/d, current smoker 10-19 cigs/d, current smoker 20+ cigs/d, unknown), histories of diabetes, hypertension and hyperlipidaemia (each yes, no, unknown), Cambridge physical activity index (inactive, moderately inactive, moderately active, active, unknown), employment status (employed or student, not employed or student, unknown), level of education completed (none or primary, secondary, vocational or university, unknown), current alcohol consumption (non-drinkers and sex-specific fifths of intake among drinkers), BMI (<22.5, 22.5-24.9, 25.0-27.4, 27.5-29.9, ≥30.0 kg/m^2^, unknown), and calibrated intakes of total energy, red and processed meat, and cheese (each continuous), and stratified by sex and EPIC centre.  ^2^ Tests of trend were performed using the calibrated intake.  ^3^ Such as tomatoes, cucumbers and sweet peppers; unavailable for Norway. 2455, 2548 and 3385 cases in never, former and current smokers, respectively.  ^4^ Unavailable for Norway and Umea. 2196, 2400 and 3174 cases in never, former and current smokers, respectively.  ^5^ Unavailable for Umea. 2198, 2411 and 3198 cases in never, former and current smokers, respectively.  ^6^ Unavailable for Denmark and Norway. 2060, 2010 and 2391 cases in never, former and current smokers, respectively.  ^7^ Tests of heterogeneity of trend by smoking status were obtained assuming independence of risk between the categories.  Abbreviations: Body mass index (BMI), ischaemic heart disease (IHD), myocardial infarction (MI). | | | | | | | | | | | |

| **Supplementary Table S6**. Hazard ratios^1^ (95% confidence intervals) for first non-fatal MI or fatal IHD per increment in calibrated intake of plant foods and fibre subdivided by age at recruitment (Whole EPIC cohort). | | | | | | | | | | | |
| --- | --- | --- | --- | --- | --- | --- | --- | --- | --- | --- | --- |
| **Food** | **Increment (g/day)** |  | **Age <55 years (2692 cases)** | |  | **Age 55-64 years (4062 cases)** | |  | **Age ≥65 years (1750 cases)** | | **P for heterogeneity^7^** |
|  |  |  | **HR (95% CI)** | **P trend^2^** |  | **HR (95% CI)** | **P trend^2^** |  | **HR (95% CI)** | **P trend^2^** |  |
| Fruit and vegetables | 200 |  | 0.98 (0.91-1.05) | 0.50 |  | 0.92 (0.86-0.98) | 0.006 |  | 0.96 (0.86-1.08) | 0.52 | 0.41 |
| Vegetables | 100 |  | 1.01 (0.91-1.12) | 0.82 |  | 0.90 (0.83-0.99) | 0.027 |  | 0.99 (0.83-1.17) | 0.90 | 0.25 |
| Fruiting vegetables^3^ | 50 |  | 0.99 (0.90-1.09) | 0.81 |  | 0.93 (0.86-1.01) | 0.090 |  | 0.93 (0.81-1.06) | 0.26 | 0.61 |
| Leafy vegetables^4^ | 50 |  | 1.14 (0.91-1.42) | 0.24 |  | 0.82 (0.67-1.01) | 0.060 |  | 1.06 (0.70-1.58) | 0.79 | 0.093 |
| Cruciferous vegetables^5^ | 50 |  | 1.13 (0.86-1.49) | 0.38 |  | 1.04 (0.86-1.26) | 0.68 |  | 1.12 (0.90-1.41) | 0.31 | 0.84 |
| Root vegetables | 50 |  | 0.85 (0.71-1.02) | 0.086 |  | 0.93 (0.83-1.05) | 0.25 |  | 0.96 (0.75-1.22) | 0.74 | 0.67 |
| Fruit | 100 |  | 0.98 (0.94-1.02) | 0.29 |  | 0.96 (0.93-1.00) | 0.042 |  | 0.99 (0.92-1.05) | 0.70 | 0.75 |
| Citrus fruit | 50 |  | 0.98 (0.93-1.04) | 0.49 |  | 0.98 (0.94-1.03) | 0.39 |  | 1.02 (0.94-1.11) | 0.64 | 0.69 |
| Apples & pears | 50 |  | 0.99 (0.95-1.02) | 0.47 |  | 0.98 (0.96-1.01) | 0.29 |  | 0.98 (0.93-1.04) | 0.56 | 0.99 |
| Banana | 50 |  | 0.93 (0.82-1.05) | 0.22 |  | 0.92 (0.84-1.01) | 0.074 |  | 0.88 (0.77-1.01) | 0.067 | 0.84 |
| Legumes^6^ | 20 |  | 1.01 (0.95-1.09) | 0.69 |  | 0.98 (0.91-1.06) | 0.67 |  | 1.05 (0.95-1.15) | 0.34 | 0.60 |
| Nuts and seeds^5^ | 10 |  | 0.90 (0.79-1.03) | 0.14 |  | 0.88 (0.77-1.02) | 0.085 |  | 1.00 (0.82-1.20) | 0.96 | 0.59 |
| Cereals and cereal products | 200 |  | 1.11 (0.91-1.34) | 0.31 |  | 0.96 (0.81-1.14) | 0.63 |  | 1.10 (0.82-1.49) | 0.52 | 0.50 |
|  |  |  |  |  |  |  |  |  |  |  |  |
| Total dietary fibre | 10 |  | 0.97 (0.86-1.10) | 0.63 |  | 0.84 (0.76-0.93) | 0.001 |  | 1.01 (0.84-1.21) | 0.94 | 0.11 |
| Fruit and vegetable fibre | 4 |  | 0.97 (0.89-1.04) | 0.36 |  | 0.93 (0.87-0.99) | 0.020 |  | 0.99 (0.88-1.11) | 0.86 | 0.56 |
| Vegetable fibre | 2 |  | 0.98 (0.87-1.09) | 0.65 |  | 0.93 (0.85-1.01) | 0.093 |  | 1.04 (0.87-1.23) | 0.68 | 0.49 |
| Fruit fibre | 2 |  | 0.98 (0.94-1.02) | 0.31 |  | 0.97 (0.93-1.00) | 0.064 |  | 0.99 (0.93-1.07) | 0.89 | 0.74 |
| Cereal fibre | 4 |  | 1.01 (0.94-1.08) | 0.89 |  | 0.95 (0.89-1.00) | 0.064 |  | 0.99 (0.88-1.11) | 0.88 | 0.42 |
| ^1^ Hazard ratios are adjusted for age (continuous), smoking status and number of cigarettes per day (never smoker, former smoker, current smoker <10 cigs/d, current smoker 10-19 cigs/d, current smoker 20+ cigs/d, unknown), histories of diabetes, hypertension and hyperlipidaemia (each yes, no, unknown), Cambridge physical activity index (inactive, moderately inactive, moderately active, active, unknown), employment status (employed or student, not employed or student, unknown), level of education completed (none or primary, secondary, vocational or university, unknown), current alcohol consumption (non-drinkers and sex-specific fifths of intake among drinkers), BMI (<22.5, 22.5-24.9, 25.0-27.4, 27.5-29.9, ≥30.0 kg/m^2^, unknown), and calibrated intakes of total energy, red and processed meat, and cheese (each continuous), and stratified by sex and EPIC centre.  ^2^ Tests of trend were performed using the calibrated intake.  ^3^ Such as tomatoes, cucumbers and sweet peppers; unavailable for Norway. 2658, 4058 and 1750 cases in participants aged <55, 55-64 and ≥65 years, respectively.  ^4^ Unavailable for Norway and Umea. 2371, 3725 and 1746 cases in participants aged <55, 55-64 and ≥65 years, respectively.  ^5^ Unavailable for Umea. 2405, 3729 and 1746 cases in participants aged <55, 55-64 and ≥65 years, respectively.  ^6^ Unavailable for Denmark and Norway. 2090, 2729 and 1713 cases in participants aged <55, 55-64 and ≥65 years, respectively.  ^7^ Tests of heterogeneity of trend by age at recruitment were obtained assuming independence of risk between the age groups.  Abbreviations: Body mass index (BMI), ischaemic heart disease (IHD), myocardial infarction (MI). | | | | | | | | | | | |

| **Supplementary Table S7.** Hazard ratios^1^ (95% confidence intervals) for first non-fatal MI or fatal IHD per increment in calibrated intake of plant foods and fibre subdivided by sex (Whole EPIC cohort). | | | | | | | | | |
| --- | --- | --- | --- | --- | --- | --- | --- | --- | --- |
| **Food** | **Increment (g/day)** | **Men** | | |  | **Women** | | | **P for heterogeneity^7^** |
|  |  | **No. of cases** | **HR (95% CI)** | **P trend^2^** |  | **No. of cases** | **HR (95% CI)** | **P trend^2^** |  |
| Fruit and vegetables | 200 | 5587 | 0.94 (0.89-0.99) | 0.015 |  | 2917 | 0.99 (0.91-1.07) | 0.78 | 0.29 |
| Vegetables | 100 | 5587 | 0.94 (0.87-1.02) | 0.12 |  | 2917 | 1.01 (0.91-1.12) | 0.80 | 0.25 |
| Fruiting vegetables^3^ | 50 | 5587 | 0.94 (0.88-1.01) | 0.097 |  | 2879 | 1.00 (0.91-1.09) | 0.92 | 0.36 |
| Leafy vegetables^4^ | 50 | 5105 | 0.89 (0.75-1.04) | 0.15 |  | 2737 | 1.30 (0.99-1.69) | 0.058 | 0.018 |
| Cruciferous vegetables^5^ | 50 | 5105 | 1.09 (0.93-1.28) | 0.28 |  | 2775 | 1.12 (0.90-1.40) | 0.31 | 0.84 |
| Root vegetables | 50 | 5587 | 0.92 (0.82-1.03) | 0.14 |  | 2917 | 0.94 (0.81-1.10) | 0.44 | 0.78 |
| Fruit | 100 | 5587 | 0.97 (0.94-1.00) | 0.041 |  | 2917 | 0.99 (0.93-1.04) | 0.60 | 0.61 |
| Citrus fruit | 50 | 5587 | 1.00 (0.96-1.03) | 0.86 |  | 2917 | 0.96 (0.89-1.02) | 0.20 | 0.30 |
| Apples & pears | 50 | 5587 | 0.98 (0.96-1.01) | 0.19 |  | 2917 | 1.00 (0.96-1.04) | 0.88 | 0.42 |
| Banana | 50 | 5587 | 0.92 (0.85-0.99) | 0.032 |  | 2917 | 0.93 (0.83-1.03) | 0.16 | 0.91 |
| Legumes^6^ | 20 | 4198 | 1.01 (0.96-1.06) | 0.66 |  | 2334 | 1.06 (0.85-1.33) | 0.58 | 0.66 |
| Nuts and seeds^5^ | 10 | 5105 | 0.91 (0.82-1.00) | 0.061 |  | 2775 | 0.87 (0.73-1.04) | 0.12 | 0.70 |
| Cereals and cereal products | 200 | 5587 | 1.09 (0.96-1.24) | 0.18 |  | 2917 | 0.85 (0.64-1.11) | 0.23 | 0.097 |
|  |  |  |  |  |  |  |  |  |  |
| Total dietary fibre | 10 | 5587 | 0.94 (0.86-1.02) | 0.16 |  | 2917 | 0.90 (0.78-1.04) | 0.15 | 0.59 |
| Fruit and vegetable fibre | 4 | 5587 | 0.94 (0.89-0.99) | 0.023 |  | 2917 | 1.00 (0.93-1.09) | 0.92 | 0.18 |
| Vegetable fibre | 2 | 5587 | 0.94 (0.87-1.02) | 0.16 |  | 2917 | 1.01 (0.91-1.12) | 0.86 | 0.31 |
| Fruit fibre | 2 | 5587 | 0.97 (0.94-1.00) | 0.053 |  | 2917 | 1.00 (0.95-1.05) | 0.99 | 0.34 |
| Cereal fibre | 4 | 5587 | 1.00 (0.95-1.05) | 0.97 |  | 2917 | 0.91 (0.82-1.00) | 0.040 | 0.063 |
| ^1^ Hazard ratios are adjusted for age (continuous), smoking status and number of cigarettes per day (never smoker, former smoker, current smoker <10 cigs/d, current smoker 10-19 cigs/d, current smoker 20+ cigs/d, unknown), histories of diabetes, hypertension and hyperlipidaemia (each yes, no, unknown), Cambridge physical activity index (inactive, moderately inactive, moderately active, active, unknown), employment status (employed or student, not employed or student, unknown), level of education completed (none or primary, secondary, vocational or university, unknown), current alcohol consumption (non-drinkers and sex-specific fifths of intake among drinkers), BMI (<22.5, 22.5-24.9, 25.0-27.4, 27.5-29.9, ≥30.0 kg/m^2^, unknown), and calibrated intakes of total energy, red and processed meat, and cheese (each continuous), and stratified by sex and EPIC centre.  ^2^ Tests of trend were performed using the calibrated intake.  ^3^ Such as tomatoes, cucumbers and sweet peppers; unavailable for Norway.  ^4^ Unavailable for Norway and Umea.  ^5^ Unavailable for Umea.  ^6^ Unavailable for Denmark and Norway.  ^7^ Tests of heterogeneity of trend by gender were obtained assuming independence of risk between men and women.  Abbreviations: Body mass index (BMI), ischaemic heart disease (IHD), myocardial infarction (MI). | | | | | | | | | |

| **Supplementary Table S8**. Hazard ratios^1^ (95% confidence intervals) for first non-fatal MI or fatal IHD per increment in calibrated intake of plant foods and fibre subdivided by BMI category (Whole EPIC cohort). | | | | | | | | | | | | | |
| --- | --- | --- | --- | --- | --- | --- | --- | --- | --- | --- | --- | --- | --- |
| **Food** | **Increment (g/day)** |  | | **BMI <25 kg/m^2^ (2602 cases)** | |  | | **BMI 25-29.9 kg/m^2^ (4049 cases)** | |  | **BMI ≥30 kg/m^2^ (1813 cases)** | | **P for heterogeneity^7^** |
|  |  |  | **HR (95% CI)** | | **P trend^2^** |  | **HR (95% CI)** | | **P trend^2^** |  | **HR (95% CI)** | **P trend^2^** |  |
| Fruit and vegetables | 200 |  | 0.92 (0.84-1.00) | | 0.050 |  | 0.94 (0.88-1.00) | | 0.038 |  | 0.97 (0.89-1.06) | 0.57 | 0.61 |
| Vegetables | 100 |  | 0.91 (0.81-1.03) | | 0.13 |  | 0.96 (0.87-1.05) | | 0.34 |  | 1.00 (0.88-1.13) | 0.97 | 0.59 |
| Fruiting vegetables^3^ | 50 |  | 0.91 (0.82-1.02) | | 0.10 |  | 0.96 (0.89-1.05) | | 0.37 |  | 0.99 (0.88-1.10) | 0.81 | 0.62 |
| Leafy vegetables^4^ | 50 |  | 1.07 (0.80-1.45) | | 0.64 |  | 0.98 (0.81-1.20) | | 0.87 |  | 0.91 (0.70-1.18) | 0.47 | 0.71 |
| Cruciferous vegetables^5^ | 50 |  | 0.96 (0.76-1.21) | | 0.72 |  | 1.25 (1.04-1.50) | | 0.017 |  | 0.91 (0.68-1.21) | 0.50 | 0.088 |
| Root vegetables | 50 |  | 0.83 (0.70-0.98) | | 0.027 |  | 0.90 (0.79-1.03) | | 0.14 |  | 1.06 (0.89-1.26) | 0.53 | 0.13 |
| Fruit | 100 |  | 0.96 (0.91-1.01) | | 0.12 |  | 0.97 (0.93-1.00) | | 0.052 |  | 0.99 (0.94-1.04) | 0.57 | 0.73 |
| Citrus fruit | 50 |  | 0.98 (0.91-1.05) | | 0.52 |  | 0.96 (0.92-1.01) | | 0.11 |  | 1.02 (0.96-1.08) | 0.57 | 0.36 |
| Apples & pears | 50 |  | 0.99 (0.95-1.03) | | 0.54 |  | 0.98 (0.95-1.01) | | 0.17 |  | 1.00 (0.96-1.04) | 0.99 | 0.71 |
| Banana | 50 |  | 0.87 (0.78-0.97) | | 0.014 |  | 0.95 (0.87-1.04) | | 0.31 |  | 0.89 (0.77-1.03) | 0.13 | 0.43 |
| Legumes^6^ | 20 |  | 1.02 (0.92-1.12) | | 0.76 |  | 0.99 (0.92-1.06) | | 0.73 |  | 1.02 (0.94-1.11) | 0.64 | 0.82 |
| Nuts and seeds^5^ | 10 |  | 0.91 (0.78-1.07) | | 0.25 |  | 0.86 (0.76-0.99) | | 0.030 |  | 0.96 (0.79-1.15) | 0.64 | 0.67 |
| Cereals and cereal products | 200 |  | 0.87 (0.70-1.08) | | 0.21 |  | 1.11 (0.94-1.31) | | 0.24 |  | 1.14 (0.88-1.47) | 0.33 | 0.17 |
|  |  |  |  | |  |  |  | |  |  |  |  |  |
| Total dietary fibre | 10 |  | 0.85 (0.75-0.97) | | 0.018 |  | 0.93 (0.84-1.04) | | 0.20 |  | 0.95 (0.81-1.11) | 0.54 | 0.47 |
| Fruit and vegetable fibre | 4 |  | 0.93 (0.86-1.01) | | 0.10 |  | 0.95 (0.89-1.01) | | 0.089 |  | 0.97 (0.88-1.06) | 0.47 | 0.84 |
| Vegetable fibre | 2 |  | 0.94 (0.84-1.06) | | 0.33 |  | 0.96 (0.88-1.06) | | 0.44 |  | 0.97 (0.85-1.10) | 0.59 | 0.95 |
| Fruit fibre | 2 |  | 0.97 (0.92-1.02) | | 0.18 |  | 0.97 (0.93-1.00) | | 0.071 |  | 0.99 (0.94-1.05) | 0.77 | 0.69 |
| Cereal fibre | 4 |  | 0.91 (0.84-0.98) | | 0.016 |  | 1.03 (0.97-1.09) | | 0.41 |  | 0.99 (0.90-1.09) | 0.83 | 0.054 |
| ^1^ Hazard ratios are adjusted for age (continuous), smoking status and number of cigarettes per day (never smoker, former smoker, current smoker <10 cigs/d, current smoker 10-19 cigs/d, current smoker 20+ cigs/d, unknown), histories of diabetes, hypertension and hyperlipidaemia (each yes, no, unknown), Cambridge physical activity index (inactive, moderately inactive, moderately active, active, unknown), employment status (employed or student, not employed or student, unknown), level of education completed (none or primary, secondary, vocational or university, unknown), current alcohol consumption (non-drinkers and sex-specific fifths of intake among drinkers), BMI (<22.5, 22.5-24.9, 25.0-27.4, 27.5-29.9, ≥30.0 kg/m^2^, unknown), and calibrated intakes of total energy, red and processed meat, and cheese (each continuous), and stratified by sex and EPIC centre.  ^2^ Tests of trend were performed using the calibrated intake.  ^3^ Such as tomatoes, cucumbers and sweet peppers; unavailable for Norway. 2578, 4039 and 1809 cases in participants with BMI <25, 25-29.9 and ≥30 kg/m^2^, respectively.  ^4^ Unavailable for Norway and Umea. 2384, 3718 and 1703 cases in participants with BMI <25, 25-29.9 and ≥30 kg/m^2^, respectively.  ^5^ Unavailable for Umea. 2408, 3728 and 1707 cases in participants with BMI <25, 25-29.9 and ≥30 kg/m^2^, respectively.  ^6^ Unavailable for Denmark and Norway. 1978, 3096 and 1418 cases in participants with BMI <25, 25-29.9 and ≥30 kg/m^2^, respectively.  ^7^ Tests of heterogeneity of trend by BMI category were obtained assuming independence of risk between categories.  Abbreviations: Body mass index (BMI), ischaemic heart disease (IHD), myocardial infarction (MI). | | | | | | | | | | | | | |

| **Supplementary Table S9.** Hazard ratios^1^ (95% confidence intervals) for first non-fatal MI or fatal IHD per increment in calibrated intake of plant foods and fibre subdivided by European region (Whole EPIC cohort). | | | | | | | | | | | |
| --- | --- | --- | --- | --- | --- | --- | --- | --- | --- | --- | --- |
| **Food** | **Increment (g/day)** |  | **Northern Europe (4387 cases)** | |  | **Central Europe (2635 cases)** | |  | **Southern Europe (1482 cases)** | | **P for heterogeneity^7^** |
|  |  |  | **HR (95% CI)** | **P trend^2^** |  | **HR (95% CI)** | **P trend^2^** |  | **HR (95% CI)** | **P trend^2^** |  |
| Fruit and vegetables | 200 |  | 0.93 (0.87-0.99) | 0.022 |  | 0.95 (0.88-1.04) | 0.29 |  | 0.96 (0.89-1.04) | 0.33 | 0.74 |
| Vegetables | 100 |  | 0.94 (0.86-1.02) | 0.15 |  | 0.91 (0.79-1.05) | 0.21 |  | 1.00 (0.89-1.12) | 0.94 | 0.60 |
| Fruiting vegetables^3^ | 50 |  | 0.95 (0.88-1.03) | 0.23 |  | 0.88 (0.78-0.99) | 0.029 |  | 1.03 (0.92-1.15) | 0.66 | 0.17 |
| Leafy vegetables^4^ | 50 |  | 1.05 (0.79-1.41) | 0.74 |  | 0.87 (0.59-1.27) | 0.46 |  | 0.99 (0.82-1.18) | 0.88 | 0.73 |
| Cruciferous vegetables^5^ | 50 |  | 1.07 (0.91-1.26) | 0.40 |  | 1.02 (0.80-1.30) | 0.89 |  | 1.43 (0.93-2.21) | 0.11 | 0.40 |
| Root vegetables | 50 |  | 0.89 (0.80-0.99) | 0.029 |  | 0.91 (0.73-1.15) | 0.44 |  | 1.03 (0.58-1.82) | 0.93 | 0.88 |
| Fruit | 100 |  | 0.96 (0.92-1.00) | 0.036 |  | 1.00 (0.95-1.05) | 1.00 |  | 0.97 (0.93-1.01) | 0.16 | 0.39 |
| Citrus fruit | 50 |  | 0.96 (0.91-1.01) | 0.15 |  | 1.00 (0.93-1.07) | 0.91 |  | 1.00 (0.95-1.06) | 0.91 | 0.50 |
| Apples & pears | 50 |  | 0.97 (0.94-1.01) | 0.12 |  | 1.03 (0.99-1.07) | 0.20 |  | 0.97 (0.94-1.01) | 0.17 | 0.092 |
| Banana | 50 |  | 0.93 (0.85-1.01) | 0.070 |  | 0.86 (0.78-0.96) | 0.006 |  | 1.09 (0.82-1.43) | 0.56 | 0.26 |
| Legumes^6^ | 20 |  | 0.76 (0.54-1.05) | 0.096 |  | 1.04 (0.98-1.11) | 0.21 |  | 0.99 (0.93-1.05) | 0.66 | 0.11 |
| Nuts and seeds^5^ | 10 |  | 0.89 (0.70-1.14) | 0.37 |  | 0.90 (0.81-1.01) | 0.080 |  | 0.92 (0.77-1.11) | 0.40 | 0.97 |
| Cereals and cereal products | 200 |  | 0.99 (0.84-1.16) | 0.89 |  | 0.97 (0.75-1.25) | 0.82 |  | 1.12 (0.88-1.42) | 0.37 | 0.67 |
|  |  |  |  |  |  |  |  |  |  |  |  |
| Total dietary fibre | 10 |  | 0.91 (0.82-1.00) | 0.054 |  | 0.91 (0.80-1.03) | 0.13 |  | 0.90 (0.74-1.10) | 0.31 | 1.00 |
| Fruit and vegetable fibre | 4 |  | 0.93 (0.88-1.00) | 0.035 |  | 0.96 (0.88-1.05) | 0.39 |  | 0.96 (0.88-1.05) | 0.40 | 0.81 |
| Vegetable fibre | 2 |  | 0.95 (0.87-1.03) | 0.21 |  | 0.94 (0.82-1.09) | 0.42 |  | 0.98 (0.86-1.11) | 0.72 | 0.92 |
| Fruit fibre | 2 |  | 0.96 (0.92-1.00) | 0.035 |  | 1.01 (0.96-1.06) | 0.83 |  | 0.97 (0.93-1.02) | 0.27 | 0.34 |
| Cereal fibre | 4 |  | 0.98 (0.93-1.03) | 0.40 |  | 0.94 (0.87-1.02) | 0.12 |  | 1.04 (0.88-1.24) | 0.61 | 0.50 |
| ^1^ Hazard ratios are adjusted for age (continuous), smoking status and number of cigarettes per day (never smoker, former smoker, current smoker <10 cigs/d, current smoker 10-19 cigs/d, current smoker 20+ cigs/d, unknown), histories of diabetes, hypertension and hyperlipidaemia (each yes, no, unknown), Cambridge physical activity index (inactive, moderately inactive, moderately active, active, unknown), employment status (employed or student, not employed or student, unknown), level of education completed (none or primary, secondary, vocational or university, unknown), current alcohol consumption (non-drinkers and sex-specific fifths of intake among drinkers), BMI (<22.5, 22.5-24.9, 25.0-27.4, 27.5-29.9, ≥30.0 kg/m^2^, unknown), and calibrated intakes of total energy, red and processed meat, and cheese (each continuous), and stratified by sex and EPIC centre.  ^2^ Tests of trend were performed using the calibrated intake.  ^3^ Such as tomatoes, cucumbers and sweet peppers; unavailable for Norway. 4349, 2635 and 1482 cases in participants in Northern, Central and Southern Europe, respectively.  ^4^ Unavailable for Norway and Umea. 3725, 2635 and 1482 cases in participants in Northern, Central and Southern Europe, respectively.  ^5^ Unavailable for Umea. 3763, 2635 and 1482 cases in participants in Northern, Central and Southern Europe, respectively.  ^6^ Unavailable for Denmark and Norway. 2415, 2635 and 1482 cases in participants in Northern, Central and Southern Europe, respectively.  ^7^ Tests of heterogeneity of trend by European region (Northern: Denmark, Norway, Sweden; Central: France excepting Provence and SW France, Germany, Netherlands, UK; Southern: Greece, Italy, Spain, Provence, SW France) were obtained assuming independence of risk between regions.  Abbreviations: Body mass index (BMI), ischaemic heart disease (IHD), myocardial infarction (MI). | | | | | | | | | | | |

| **Supplementary Table S10.** Hazard ratios^1^ (95% confidence intervals) for first non-fatal MI or fatal IHD per increment in calibrated intake of plant foods and fibre, stratified by prior disease status (histories of diabetes, hypertension and hyperlipidaemia) (Whole EPIC cohort). | | | | | | |
| --- | --- | --- | --- | --- | --- | --- |
|  | **Prior disease** | | | | | |
|  | **No** | |  | **Yes** | |  |
| **Foods, g/day** | **Cases** | **HR (95% CI)** |  | **Cases** | **HR (95% CI)** | **P for heterogeneity^6^** |
| Fruit and vegetables | 2884 | 0.95 (0.90 - 1.01) |  | 3607 | 0.93 (0.89 - 0.99) | 0.591 |
| Vegetables | 2884 | 0.96 (0.88 - 1.05) |  | 3607 | 0.95 (0.88 - 1.03) | 0.768 |
| Fruiting vegetables^2^ | 2884 | 0.94 (0.87 - 1.01) |  | 3594 | 0.96 (0.89 - 1.02) | 0.638 |
| Leafy vegetables^3^ | 2505 | 0.98 (0.81 - 1.17) |  | 3350 | 0.96 (0.82 - 1.12) | 0.814 |
| Cruciferous vegetables^4^ | 2505 | 1.22 (0.99 - 1.51) |  | 3363 | 1.13 (0.94 - 1.35) | 0.486 |
| Root vegetables | 2884 | 0.96 (0.83 - 1.12) |  | 3607 | 0.94 (0.83 - 1.07) | 0.824 |
| Fruit | 2884 | 0.97 (0.94 - 1.01) |  | 3607 | 0.96 (0.93 - 0.99) | 0.577 |
| Citrus fruit | 2884 | 1.01 (0.97 - 1.06) |  | 3607 | 0.97 (0.93 - 1.01) | 0.156 |
| Apples & pears | 2884 | 1.00 (0.97 - 1.03) |  | 3607 | 0.97 (0.94 - 1.00) | 0.189 |
| Banana | 2884 | 0.86 (0.78 - 0.95) |  | 3607 | 0.89 (0.81 - 0.98) | 0.631 |
| Legumes^5^ | 2212 | 1.04 (0.99 - 1.09) |  | 2985 | 0.98 (0.93 - 1.04) | 0.056 |
| Nuts and seeds^4^ | 2505 | 0.84 (0.75 - 0.94) |  | 3363 | 0.93 (0.83 - 1.03) | 0.107 |
| Cereals and cereal products | 2884 | 0.95 (0.83 - 1.10) |  | 3607 | 1.03 (0.90 - 1.17) | 0.285 |
| Total dietary fibre | 2884 | 0.96 (0.88 - 1.05) |  | 3607 | 0.90 (0.83 - 0.98) | 0.202 |
| Fruit and vegetable fibre | 2884 | 0.96 (0.90 - 1.02) |  | 3607 | 0.94 (0.89 - 1.00) | 0.571 |
| Vegetable fibre | 2884 | 0.97 (0.89 - 1.06) |  | 3607 | 0.96 (0.88 - 1.03) | 0.738 |
| Fruit fibre | 2884 | 0.98 (0.94 - 1.01) |  | 3607 | 0.97 (0.94 - 1.00) | 0.688 |
| Cereal fibre | 2884 | 0.98 (0.93 - 1.03) |  | 3607 | 0.98 (0.93 - 1.03) | 0.930 |
| ^1^ Hazard ratios are adjusted for age (continuous), smoking status and number of cigarettes per day (never smoker, former smoker, current smoker <10 cigs/d, current smoker 10-19 cigs/d, current smoker 20+ cigs/d, unknown), Cambridge physical activity index (inactive, moderately inactive, moderately active, active, unknown), employment status (employed or student, not employed or student, unknown), level of education completed (none or primary, secondary, vocational or university, unknown), current alcohol consumption (non-drinkers and sex-specific fifths of intake among drinkers), BMI (<22.5, 22.5-24.9, 25.0-27.4, 27.5-29.9, ≥30.0 kg/m^2^, unknown), and calibrated intakes of total energy, red and processed meat, and cheese (each continuous), and stratified by sex and EPIC centre.  ^2^ Such as tomatoes, cucumbers and sweet peppers; unavailable for Norway  ^3^ Unavailable for Norway and Umea.  ^4^ Unavailable for Umea.  ^5^ Unavailable for Denmark and Norway.  ^6^ Tests of heterogeneity of trend by prior disease status were obtained assuming independence of risk by prior disease status using a meta-analysis method.  Abbreviations: Body mass index (BMI), ischaemic heart disease (IHD), myocardial infarction (MI). | | | | | | |

| **Supplementary Table S11.** Associations of fruit, vegetables, legumes, and nuts and seeds with potential mediators in non-cases (Whole EPIC cohort and EPIC-CVD study for biomarker data) | | | | | | | |
| --- | --- | --- | --- | --- | --- | --- | --- |
|  | **Number of observations** | **Overall fifth of intake** | | | | | **P trend^1^** |
|  |  | **Lowest** | **2** | **Middle** | **4** | **Highest** |  |
| **Fruit and vegetables** |  |  |  |  |  |  |  |
| Body mass index (kg/m^2^) | 477,859 | 25.37 (25.35-25.40) | 25.39 (25.36-25.42) | 25.42 (25.40-25.45) | 25.54 (25.51-25.56) | 25.70 (25.67-25.73) | <0.0001 |
| Systolic blood pressure (mmHg) | 306,829 | 131.6 (131.5-131.8) | 131.1 (131.0-131.2) | 130.8 (130.7-131.0) | 130.9 (130.7-131.0) | 130.6 (130.5-130.8) | <0.0001 |
| Diastolic blood pressure (mmHg) | 306,808 | 81.2 (81.1-81.3) | 80.9 (80.9-81.0) | 80.8 (80.8-80.9) | 80.9 (80.8-80.9) | 80.8 (80.7-80.9) | <0.0001 |
| Total cholesterol (mmol/L)^2^ | 16,425 | 6.02 (5.98–6.06) | 5.96 (5.92–6.00) | 5.91 (5.87–5.95) | 5.94 (5.90–5.98) | 5.89 (5.85–5.93) | 0.0002 |
| HDL cholesterol (mmol/L)^2^ | 16,425 | 1.47 (1.46–1.49) | 1.49 (1.48–1.51) | 1.49 (1.48–1.50) | 1.48 (1.47–1.50) | 1.49 (1.48–1.51) | 0.23 |
| non-HDL cholesterol (mmol/L)^2^ | 16,421 | 4.55 (4.51–4.59) | 4.47 (4.43–4.51) | 4.42 (4.38–4.46) | 4.46 (4.42–4.50) | 4.39 (4.35–4.44) | 0.0001 |
| HbA1c | 16,812 | 5.51 (5.49-5.53) | 5.51 (5.49-5.53) | 5.51 (5.49-5.53) | 5.52 (5.50-5.54) | 5.54 (5.52-5.56) | 0.041 |
|  |  |  |  |  |  |  |  |
| **Fruit** |  |  |  |  |  |  |  |
| Body mass index (kg/m^2^) | 477,859 | 25.44 (25.41-25.46) | 25.44 (25.42-25.47) | 25.45 (25.43-25.48) | 25.50 (25.47-25.52) | 25.59 (25.56-25.62) | <0.0001 |
| Systolic blood pressure (mmHg) | 306,829 | 131.4 (131.2-131.5) | 131.0 (130.9-131.2) | 130.9 (130.8-131.1) | 131.1 (130.9-131.2) | 130.8 (130.6-130.9) | <0.0001 |
| Diastolic blood pressure (mmHg) | 306,808 | 81.1 (81.0-81.2) | 80.9 (80.8-81.0) | 80.9 (80.8-81.0) | 81.0 (80.9-81.1) | 80.8 (80.7-80.9) | 0.001 |
| Total cholesterol (mmol/L)^2^ | 16,425 | 6.03 (5.99-6.07) | 5.94 (5.90-5.98) | 5.93 (5.90-5.97) | 5.92 (5.88-5.96) | 5.90 (5.86-5.93) | 0.0001 |
| HDL cholesterol (mmol/L)^2^ | 16,425 | 1.48 (1.47-1.50) | 1.50 (1.48-1.51) | 1.49 (1.48-1.51) | 1.48 (1.46-1.49) | 1.48 (1.47-1.50) | 0.42 |
| non-HDL cholesterol (mmol/L)^2^ | 16,421 | 4.55 (4.51-4.59) | 4.44 (4.40-4.48) | 4.44 (4.40-4.48) | 4.44 (4.41-4.48) | 4.41 (4.37-4.45) | 0.0004 |
| HbA1c | 16,812 | 5.52 (5.50-5.54) | 5.51 (5.49-5.53) | 5.51 (5.49-5.53) | 5.51 (5.49-5.53) | 5.53 (5.51-5.55) | 0.31 |
|  |  |  |  |  |  |  |  |
| **Vegetables** |  |  |  |  |  |  |  |
| Body mass index (kg/m^2^) | 477,859 | 25.37 (25.34-25.39) | 25.38 (25.36-25.41) | 25.42 (25.39-25.44) | 25.52 (25.49-25.54) | 25.73 (25.71-25.76) | <0.0001 |
| Systolic blood pressure (mmHg) | 306,829 | 131.7 (131.6-131.9) | 131.3 (131.1-131.4) | 130.9 (130.8-131.0) | 130.7 (130.5-130.8) | 130.4 (130.2-130.5) | <0.0001 |
| Diastolic blood pressure (mmHg) | 306,808 | 81.2 (81.1-81.3) | 81.1 (81.0-81.1) | 80.9 (80.8-80.9) | 80.8 (80.7-80.8) | 80.7 (80.6-80.8) | <0.0001 |
| Total cholesterol (mmol/L)^2^ | 16,425 | 5.98 (5.94-6.02) | 5.95 (5.91-5.99) | 5.94 (5.90-5.97) | 5.93 (5.90-5.97) | 5.91 (5.87-5.95) | 0.040 |
| HDL cholesterol (mmol/L) ^2^ | 16,425 | 1.47 (1.45-1.48) | 1.47 (1.46-1.49) | 1.49 (1.48-1.50) | 1.49 (1.48-1.51) | 1.51 (1.49-1.52) | 0.0002 |
| non-HDL cholesterol (mmol/L)^2^ | 16,421 | 4.52 (4.48-4.56) | 4.48 (4.44-4.52) | 4.45 (4.41-4.48) | 4.44 (4.40-4.48) | 4.41 (4.36-4.45) | 0.001 |
| HbA1c | 16,812 | 5.52 (5.50-5.54) | 5.52 (5.50-5.54) | 5.51 (5.49-5.53) | 5.52 (5.50-5.54) | 5.53 (5.51-5.55) | 0.50 |
|  |  |  |  |  |  |  |  |
| **Legumes**^3^ |  |  |  |  |  |  |  |
| Body mass index (kg/m^2^) | 391,001 | 25.78 (25.74-25.81) | 25.75 (25.71-25.78) | 25.79 (25.76-25.83) | 25.89 (25.85-25.92) | 25.89 (25.85-25.92) | <0.0001 |
| Systolic blood pressure (mmHg) | 255,499 | 130.0 (129.8-130.2) | 129.9 (129.7-130.1) | 130.2 (130.0-130.4) | 130.5 (130.3-130.7) | 130.2 (130.0-130.4) | 0.037 |
| Diastolic blood pressure (mmHg) | 255,477 | 80.5 (80.3-80.6) | 80.4 (80.3-80.5) | 80.6 (80.5-80.8) | 80.7 (80.6-80.9) | 80.6 (80.4-80.7) | 0.11 |
|  |  |  |  |  |  |  |  |
| **Nuts and seeds** |  |  |  |  |  |  |  |
| Body mass index (kg/m^2^) | 477,859 | 25.64 (25.61-25.66) | 25.67 (25.64-25.70) | 25.47 (25.44-25.50) | 25.41 (25.38-25.43) | 25.23 (25.20-25.26) | <0.0001 |
| Systolic blood pressure (mmHg) | 306,829 | 131.9 (131.7-132.0) | 131.0 (130.8-131.2) | 131.1 (131.0-131.2) | 130.7 (130.6-130.9) | 130.3 (130.1-130.4) | <0.0001 |
| Diastolic blood pressure (mmHg) | 306,808 | 81.1 (81.0-81.2) | 81.1 (81.0-81.2) | 80.9 (80.8-81.0) | 80.9 (80.8-81.0) | 80.6 (80.5-80.7) | <0.0001 |
| Total cholesterol (mmol/L)^2^ | 16,425 | 5.94 (5.91-5.98) | 5.95 (5.88-6.01) | 5.96 (5.92-6.00) | 5.96 (5.92-6.00) | 5.91 (5.87-5.95) | 0.11 |
| HDL cholesterol (mmol/L) ^2^ | 16,425 | 1.47 (1.45-1.48) | 1.49 (1.47-1.51) | 1.48 (1.47-1.50) | 1.50 (1.49-1.52) | 1.50 (1.49-1.52) | 0.003 |
| non-HDL cholesterol (mmol/L)^2^ | 16,421 | 4.48 (4.44-4.51) | 4.45 (4.39-4.52) | 4.48 (4.43-4.52) | 4.46 (4.42-4.50) | 4.41 (4.37-4.45) | 0.012 |
| HbA1c | 16,812 | 5.53 (5.52-5.55) | 5.54 (5.50-5.57) | 5.50 (5.48-5.52) | 5.52 (5.50-5.54) | 5.50 (5.48-5.52) | 0.062 |
|  |  |  |  |  |  |  |  |
| **Cereals and cereal products**^3^ |  |  |  |  |  |  |  |
| Body mass index (kg/m^2^) | 477,859 | 25.65 (25.62-25.67) | 25.63 (25.61-25.66) | 25.50 (25.48-25.53) | 25.40 (25.35-25.40) | 25.26 (25.23-25.29) | <0.0001 |
| Systolic blood pressure (mmHg) | 306,829 | 131.1 (131.0-131.3) | 131.2 (131.0-131.3) | 131.1 (131.0-131.3) | 131.0 (130.9-131.2) | 130.7 (130.6-130.9) | <0.0001 |
| Diastolic blood pressure (mmHg) | 306,808 | 81.3 (81.2-81.4) | 81.1 (81.0-81.2) | 81.0 (80.9-81.1) | 80.8 (80.7-80.9) | 80.5 (80.4-80.6) | <0.0001 |
|  |  |  |  |  |  |  |  |
| **Dietary fibre** |  |  |  |  |  |  |  |
| Body mass index (kg/m^2^) | 477,859 | 25.55 (25.53-25.58) | 25.57 (25.54-25.59) | 25.49 (25.47-25.52) | 25.46 (25.44-25.49) | 25.35 (25.32-25.37) | <0.0001 |
| Systolic blood pressure (mmHg) | 306,829 | 131.6 (131.4-131.7) | 131.3 (131.2-131.4) | 131.1 (130.9-131.2) | 130.8 (130.7-131.0) | 130.4 (130.2-130.5) | <0.0001 |
| Diastolic blood pressure (mmHg) | 306,808 | 81.3 (81.3-81.4) | 81.2 (81.1-81.2) | 81.0 (81.0-81.1) | 80.8 (80.7-80.9) | 80.3 (80.3-80.4) | <0.0001 |
| Total cholesterol (mmol/L)^2^ | 16,425 | 6.01 (5.97–6.05) | 5.99 (5.95–6.03) | 5.93 (5.89–5.96) | 5.92 (5.89–5.96) | 5.87 (5.83–5.91) | <0.0001 |
| HDL cholesterol (mmol/L) ^2^ | 16,425 | 1.48 (1.47–1.49) | 1.51 (1.49–1.52) | 1.48 (1.47–1.49) | 1.48 (1.47–1.50) | 1.48 (1.47–1.49) | 0.34 |
| non-HDL cholesterol (mmol/L)^2^ | 16,421 | 4.53 (4.49–4.57) | 4.48 (4.45–4.52) | 4.45 (4.41–4.48) | 4.44 (4.40–4.48) | 4.39 (4.35–4.43) | <0.0001 |
| HbA1c | 16,812 | 5.50 (5.48-5.52) | 5.52 (5.50-5.54) | 5.52 (5.50-5.53) | 5.53 (5.51-5.55) | 5.53 (5.51-5.55) | 0.10 |
| Values are means (95% CI) and have been adjusted for age, sex and EPIC-CVD centre.  ^1^ The tests of trend are based on the median intake in each category of the overall fifths of intake. For fruit and vegetables the median intakes within each category are: 163, 278, 391, 531 and 791 g/day; for fruit the median intakes within each category are: 52, 123, 194, 283 and 452 g/day; for vegetables, the median intakes are: 70, 122, 176, 251 and 399 g/day; for legumes, the median intakes are: 0.0, 2.7, 8.6, 18.1 and 45.2 g/day; for nuts and seeds, the median intakes are: 0.00, 0.26, 0.82, 3.19 and 10.96 g/day; and for dietary fibre, the median intakes are: 13.9, 18.3, 21.8, 25.7 and 32.7 g/day.  ^2^ Restricted to a sub-cohort randomly selected from participants with a stored blood sample.]  ^3^ Legume and cereals intake data not available in EPIC-CVD subsample with biomarker data.  Abbreviations: Body mass index (BMI), Hemoglobin A1c (HbA1c), ischaemic heart disease (IHD), myocardial infarction (MI). | | | | | | | |
